# Transcriptional regulation of a gonococcal gene encoding a virulence factor (L-lactate permease)

**DOI:** 10.1371/journal.ppat.1008233

**Published:** 2019-12-20

**Authors:** Julio C. Ayala, William M. Shafer

**Affiliations:** 1 Department of Microbiology and Immunology, Emory University School of Medicine, Atlanta, Georgia, United States of America; 2 The Emory Antibiotic Resistance Center, Emory University School of Medicine, Atlanta, Georgia, United States of America; 3 Laboratories of Bacterial Pathogenesis, Veterans Affairs Medical Center, Decatur, Georgia, United States of America; University of Oxford, UNITED KINGDOM

## Abstract

GdhR is a GntR-type regulator of *Neisseria gonorrhoeae* encoded by a gene (*gdhR*) belonging to the MtrR regulon, which comprises multiple genes required for antibiotic resistance such as the *mtrCDE* efflux pump genes. In previous work we showed that loss of *gdhR* results in enhanced gonococcal fitness in a female mouse model of lower genital tract infection. Here, we used RNA-Seq to perform a transcriptional profiling study to determine the GdhR regulon. GdhR was found to regulate the expression of 2.3% of all the genes in gonococcal strain FA19, of which 39 were activated and 11 were repressed. Within the GdhR regulon we found that *lctP*, which encodes a unique L-lactate transporter and has been associated with gonococcal pathogenesis, was the highest of GdhR-repressed genes. By using *in vitro* transcription and DNase I footpriting assays we mapped the *lctP* transcriptional start site (TSS) and determined that GdhR directly inhibits transcription by binding to an inverted repeat sequence located 9 bases downstream of the *lctP* TSS. Epistasis analysis revealed that, while loss of *lctP* increased susceptibility of gonococci to hydrogen peroxide (H_2_O_2_) the loss of *gdhR* enhanced resistance; however, this GdhR-endowed property was reversed in a double *gdhR lctP* null mutant. We assessed the effect of different carbon sources on *lctP* expression and found that D-glucose, but not L-lactate or pyruvate, repressed *lctP* expression within a physiological concentration range but in a GdhR-independent manner. Moreover, we found that adding glucose to the medium enhanced susceptibility of gonococci to hydrogen peroxide. We propose a model for the role of *lctP* regulation via GdhR and glucose in the pathogenesis of *N*. *gonorrhoeae*.

## Author summary

LctP is a unique lactate transporter encoded in the genome of *N*. *gonorrhoeae* and lactate is one of the few carbon energy sources that can be used by pathogenic *Neisseria*. Lactate acquisition has been shown to increase gonococcal resistance to complement-mediated killing by human serum and to enhance colonization and survival in a female mouse model of lower genital tract infection. Herein, we describe the transcriptional regulation of *lctP* in gonococci. We showed that the GdhR regulator and D-glucose act independently to dampen *lctP* expression and that such regulation has an impact on the resistance of gonococcal cells to killing by hydrogen peroxide. Thus, this study provides insights regarding the mechanism by which gonococci survive during infection and identify a new virulence phenotype linked to *lctP* gene expression.

## Introduction

Gonorrhea is a sexual transmitted infection (STI) caused by the Gram-negative bacterium *Neisseria gonorrhoeae*. Gonorrhea is the second most common bacterial STI with a burden in the US alone of 468,514 reported cases [1] and an estimated 86.9 million cases worldwide in 2016 [2]. Since 1938 the gonococcus has developed resistance to all the antibiotics that have been introduced in the clinic to treat this STI, including recent cases of resistance to the currently used dual antibiotic treatment regimen of ceftriaxone and azithromycin [3]. Among the mechanisms developed by *N*. *gonorrhoeae* strains to acquire antimicrobial resistance are the re-modeling of the beta-lactam lethal target, penicillin-binding protein 2 (PBP2) due to acquisition of mutations within *penA* [4] or formation of a mosaic *penA* due to recombination of donated DNA sequences from commensal Neisseria [5,6]; amino acid replacements in the major porin protein encoded by *porB*, limiting the influx of antibiotics into the cell [7]; and the increased expression of the antimicrobial efflux pump MtrCDE due to transcriptional regulatory mutations impacting expression of the *mtrR* gene, which encodes the master repressor of *mtrCDE* [8].

MtrR is a global regulator of gonococci that belongs to the TetR-family and has a central role in pathogenesis and antibiotic resistance [9,10]. Its regulon extends beyond the *mtr* locus and comprises multiple genes required for pathogenesis and stress response such as the alternative sigma factor *rpoH* [9]. In this work we focused on one gene within the MtrR regulon, *gdhR*, encoding the GntR-type transcriptional regulator GdhR. The gene is located immediately downstream the *mtrCDE* locus and is subjected to MtrR transcriptional repression [11]. In previous work we showed that loss of GdhR results in enhanced gonococcal fitness in a female mouse model of lower genital tract infection [11]. However, the GdhR-regulated genes responsible for this *in vivo* phenotype remained unknown.

Most of the regulatory functions of GdhR have been studied in the closely related pathogen *N*. *meningitidis* that, unlike gonococci, can be carried commensally in many people [12]. In meningococci, GdhR mediates a growth phase- and carbon source-dependent positive regulation of *gdhA* encoding an L-glutamate dehydrogenase, a gene required for systemic infection in an infant rat model [13]; however in gonococci the corresponding *gdhA* homologue was not found to be regulated by GdhR [11]. This suggested that, despite the high degree of DNA sequence conservation in both gonococci and meningococci, their regulatory circuits do not share similar functions due to changes in non-coding regions such as promoter sequences [11]. In addition to *gdhA*, meningococcal GdhR was found to regulate several genes involved in glucose catabolism by the Entner-Doudoroff pathway and in L-glutamate import [14]. Similarly, genes within the GntR-family regulate different biological processes including the oxidation of substrates such as pyruvate (PdhR), lactate (LldR) or gluconate (GntR, the family founder) [15,16]. An important feature of these regulators is the presence of an N-terminal helix-turn-helix (HTH) DNA binding domain and a C-terminal metabolite-binding and oligomerization domain [15,17]. The HTH domain is highly conserved among the family members, while the oligomerization domain is less conserved and can regulate the DNA-binding activity of the HTH domain by imposing steric constraints that influences protein mobility [18].

In this study we performed a transcriptomic analysis to determine the gonococcal GdhR regulon to help understand the mechanism by which loss of GdhR enhances the *in vivo* fitness and survival of gonococci during lower genital tract infection of female mice [11]. In this respect, we present evidence that GdhR is a direct repressor of *lctP* expression. The gene *lctP* encodes a unique L-lactate permease in the genome of gonococcal cells and has been linked to pathogenesis [19]. Previous work showed that gonococcal *lctP* null mutants had a growth defect in medium containing physiological concentrations of glucose and lactate, were more susceptible to killing by normal human serum and were significantly impaired for colonization and survival in the female mice model of infection [19]. In general, lactate derived from host cells enhances gonococcal metabolism and sialylation of the lipooligosaccharide (LOS), induces serum resistance and increases survival in human polymorphonuclear leukocytes and cervical epithelial cells [20–22]. Further, we show that GdhR regulation of *lctP* gene expression, as well as the presence of glucose in the medium, can independently influence the overall resistance of gonococci to hydrogen peroxide.

## Results

### Transcriptional modulation of a gonococcal virulence factor-encoding gene (*lctP*) by GdhR

To determine the expression levels of *gdhR* (NGO1360 locus tag in the FA1090 reference strain) at different points of the growth curve we collected total RNA from *N*. *gonorrhoeae* strain FA19 grown in GC broth at different optical densities. The levels of *gdhR* mRNA were determined by qRT-PCR along with the levels of its direct repressor (MtrR) encoded by *mtrR* (NGO1366) and the MtrR-regulated gene *mtrC* (NGO1365). As a control we used *rmpM* (NGO1577) encoding a highly expressed and conserved outer membrane protein antigen [23]. The results showed that the expression levels of *mtrR* or two of its regulated genes (*gdhR* and *mtrC*) did not significantly change between the exponential and stationary phases of growth (S1 Fig).

**Fig 1:**
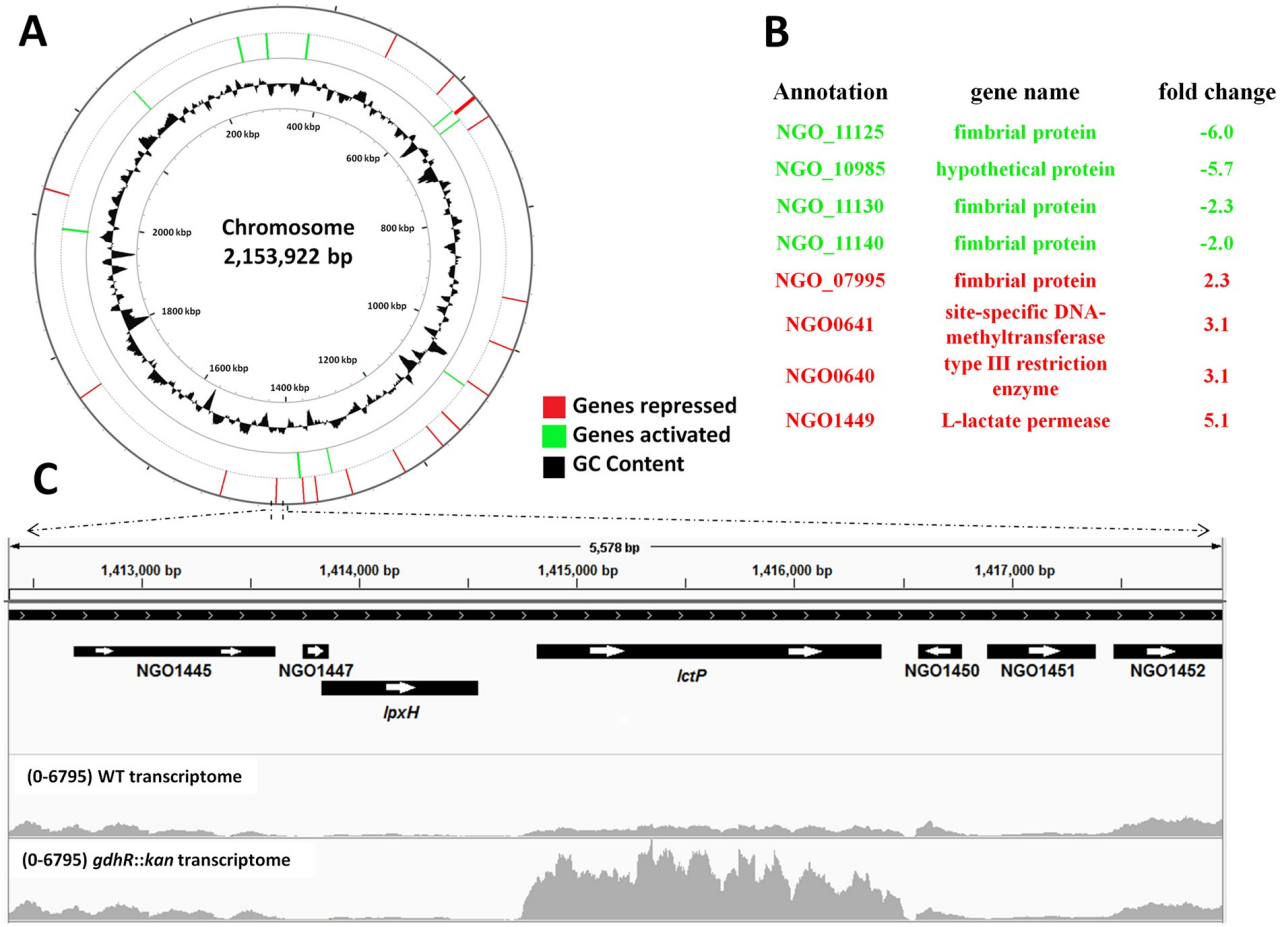
Genome-wide GdhR regulon during late-exponential growth. A. Graphic representation generated by CG viewer [67], depicting the *Neisseria gonorrhoeae* chromosome showing GdhR-regulated genes determined by RNA-Seq. *N*. *gonorrhoeae* cells were grown in GC broth at 37°C and collected at late-exponential phase for RNA-Seq. The numbering indicated within the inner circle represents the chromosome coordinates in kb pairs. B. List of GdhR-repressed and -activated protein-coding genes compiled at a fold change higher than 2. C. Transcription profile of the *lctP* locus in FA19 WT and *gdhR* mutant gonococci. BAM files containing the sequence reads for the WT (GEO sample number GSM3981651) and *gdhR*::*kan* (GSM3981653) cells transcriptomes were aligned to the FA19 strain genome and visualized using IGV software [68]. Genes are represented using the FA1090 annotation system. Numbers in parenthesis indicate the maximum read count in the (y) axis.

To determine the GdhR regulon an RNA-Seq analysis was performed using total RNA samples collected from wild-type (WT) strain FA19 and its isogenic *gdhR* insertional mutant (FA19 *gdhR*::*kan*) grown to late-exponential phase in GC broth. The number of genes that were differentially regulated by GdhR represented 2.3% of all the genes in the FA19 strain genome (Fig 1A). Of the GdhR-regulated genes, 39 were activated and 11 were repressed (S1 Table). However, the list of differentially expressed genes in WT versus *gdhR* mutant compiled at a fold-change > 2 contained only 11 genes, of which only 8 corresponded to protein coding genes (Fig 1B). Anticipating that GdhR could be a cryptic regulator we also included a comparison of the transcriptomes of *gdhR* mutant cells and a complemented mutant in which *gdhR* expression signals are bypassed by overexpression from the inducible *lac* promoter in vector pGCC4 (strain JC01). This analysis showed that under conditions of overexpression, GdhR differentially regulated 46 genes (S2 Table), out which 12 were the same genes regulated in the WT background (S1 Table). These results suggested that GdhR is not a cryptic regulator in the GC broth-grown WT background, since it can regulate at least 12 identical genes under conditions of WT and artificial overexpression of GdhR levels. Accordingly, we focused on the WT background GdhR regulon, which included mostly genes annotated as fimbrial proteins and membrane transporters (Fig 1B and S1 Table). We also noted that *lctP* (NGO1449) was the highest of GdhR-repressed genes (Fig 1B and 1C).

**Fig 2:**
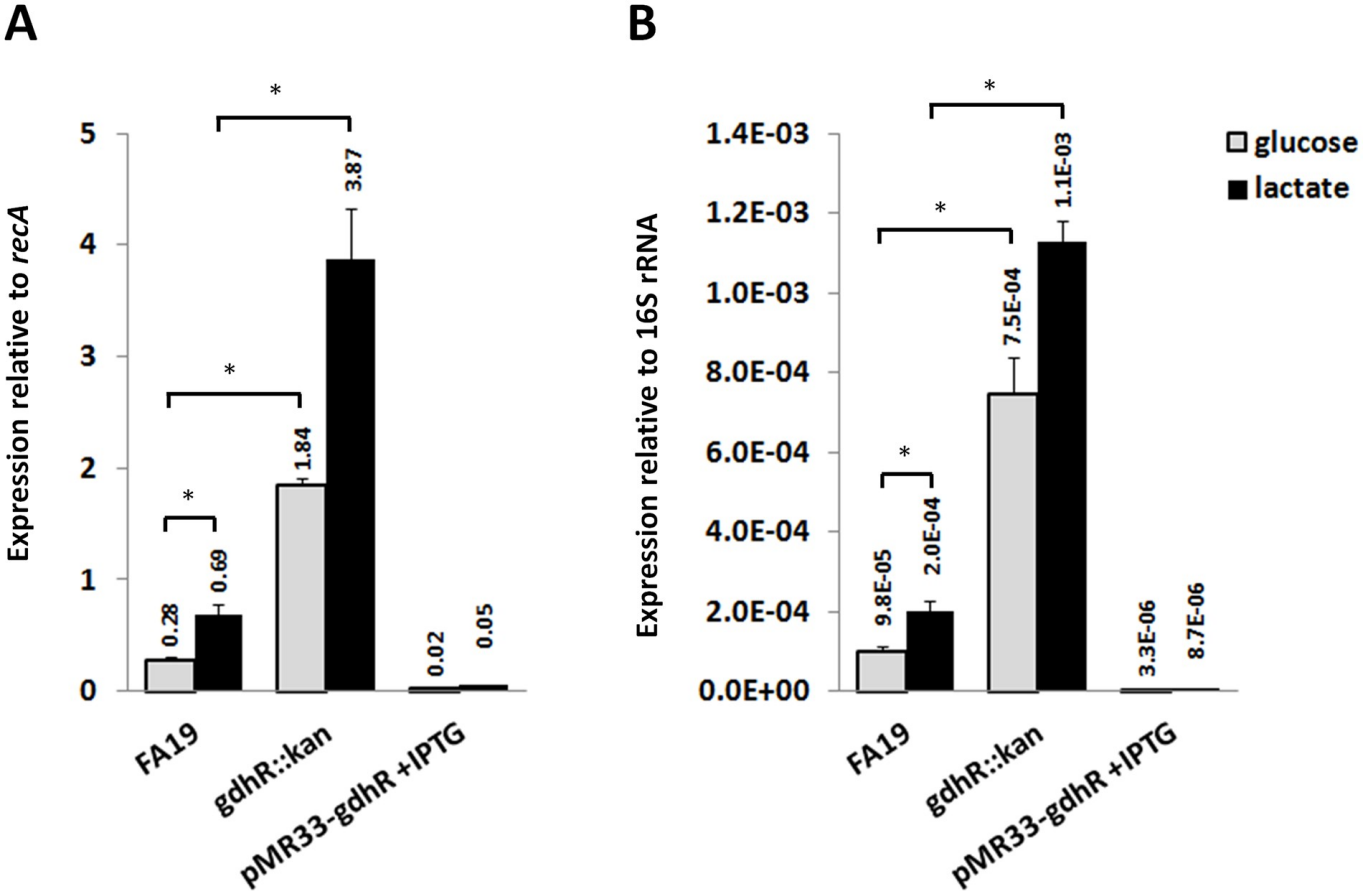
Regulation of the *lctP* allele by GdhR. Relative levels of *lctP* mRNA were determined by qRT-PCR using *recA* (A) or 16S rRNA (B) as internal reference in total RNA samples from WT strain FA19 and its isogenic mutants *gdhR*::*kan* and *gdhR*::*kan* complemented strain JC02 (pMR33-*gdhR*). Cells were grown to late-logarithmic phase in GC broth supplemented with either glucose (grey bars) or lactate (black) at 22 mM each. Data are presented as the mean (bar) plus the standard error of the mean (error bar) of 3 biological samples. * represents significant statistical differences at p<0.05 as determined by a one-tailed non-parametric Mann Whitney U-test.

**Fig 3:**
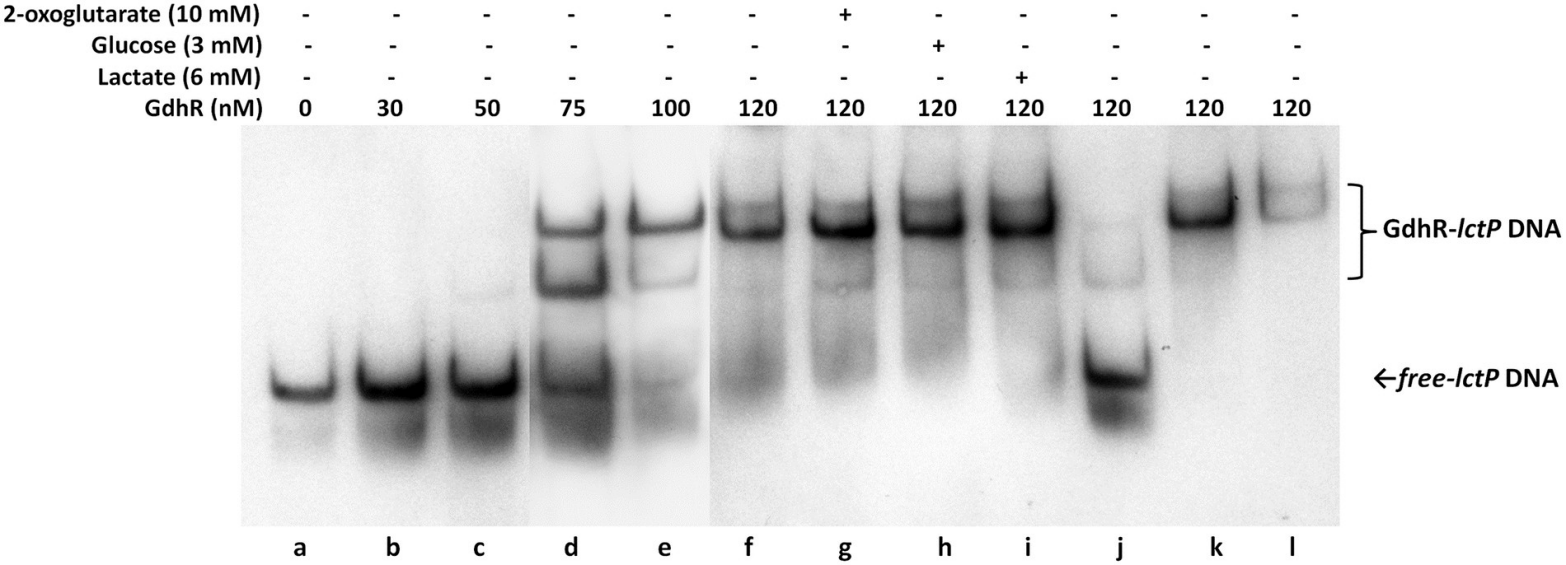
Binding of GdhR to the *lctP* promoter region. A DIG-labeled DNA fragment spanning nucleotides −313 to −23 of the *lctP* promoter relative to the start codon was incubated with increasing concentrations of purified GdhR. The mobility of free DNA and of the nucleoprotein complexes were determined by EMSA and are indicated at the right of the gel. Competitive EMSAs were prepared by adding a 100-fold excess of unlabeled DNA fragments encoding *lctP* and *recA* promoter regions (lane j and k respectively) and 16S ribosomal RNA (lane l).

Because of the recognized importance of *lctP* and lactate transport and utilization for gonococcal pathogenesis (reviewed in [24,25]), we focused on GdhR regulation of *lctP*. qRT-PCR analysis was performed to validate the RNA-Seq results. The results showed that *lctP* was repressed 7-fold by GdhR (Fig 2A). Similarly, under conditions of GdhR overexpression from an ectopic promoter *lctP* could be further repressed at 92-fold. To determine whether GdhR regulation of *lctP* is direct we conducted electrophoresis mobility shift assays (EMSA). We used the transcriptomic files of the RNA-Seq analysis to estimate the approximate location of the *lctP* TSS and promoter region (Fig 1C). We found that purified GdhR could bind a DNA fragment spanning the *lctP* promoter region (Fig 3). This binding was specific since an excess of unlabeled *lctP* promoter DNA completely competed with the labeled *lctP* DNA (Fig 3, lane j) while an excess of unlabeled DNAs encoding non-GdhR-regulated housekeeping genes could not (Fig 3 lanes k and l).

To test whether GdhR represses *lctP* in a different strain background we inactivated the *gdhR* gene in laboratory strain F62 and conducted qRT-PCR. The results revealed that GdhR also repressed *lctP* expression in F62 (S2 Fig). An alignment of the *lpxH* (NGO1448)-*lctP* intergenic region, containing the *lctP* promoter (Fig 1C), revealed identical sequences with only one nucleotide polymorphism among different laboratory strains and recent clinical isolates (S3 Fig). This and the high degree of conservation of the *gdhR* allele and its upstream region among different gonococcal strains (99.7%, S1 Appendix) suggests that GdhR regulation of *lctP* is likely to be a conserved trait among gonococci strains.

### Molecular mechanism of *lctP* repression by GdhR

**Fig 4:**
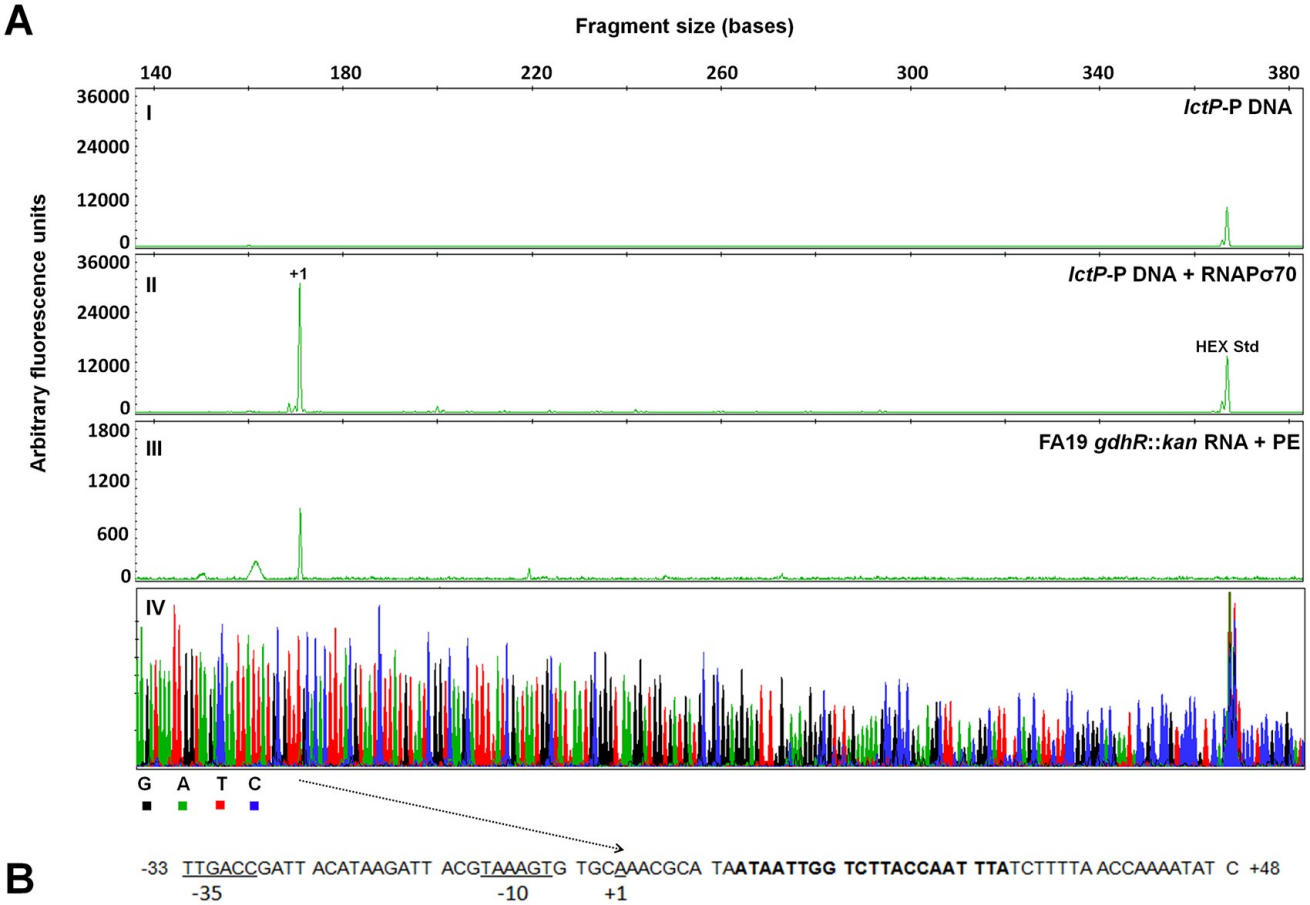
Mapping of the *lctP* transcriptional start site by *in vitro* transcription and primer extension (PE). A. A *lctP* DNA fragment spanning nucleotides -192 to +381 relative to the TSS was incubated with (II) or without (I) purified RNAPσ70. The resulting RNA transcript (+1) and total RNA from *gdhR*::*kan* mutant cells (III) were purified and annealed with primer HEX-lctP-IvT complimentary to the *lctP* coding sequence and extended as described in *Methods*. The resulting fluorescent peaks were analyzed in a capillary electrophoresis sequencer. A HEX-labeled 369 bp DNA standard (HEX Std) was added to *in vitro* transcription reactions before analysis. Manually-generated sequencing reactions (IV) were obtained with primer HEX-lctP-IvT and the above *lctP* DNA template. GeneMapper was used to align the resulting electropherograms, to estimate the height (y axis) and the size (x axis at the top) of the peaks and to obtain a color-coded overlay representation of the template strand sequence (IV). B. Architecture of the *lctP* promoter showing the mapped TSS (+1) and sigma-70 promoter elements underlined. An inverted repeat matching the consensus FadR DNA-binding motif is represented in bold font.

**Fig 5:**
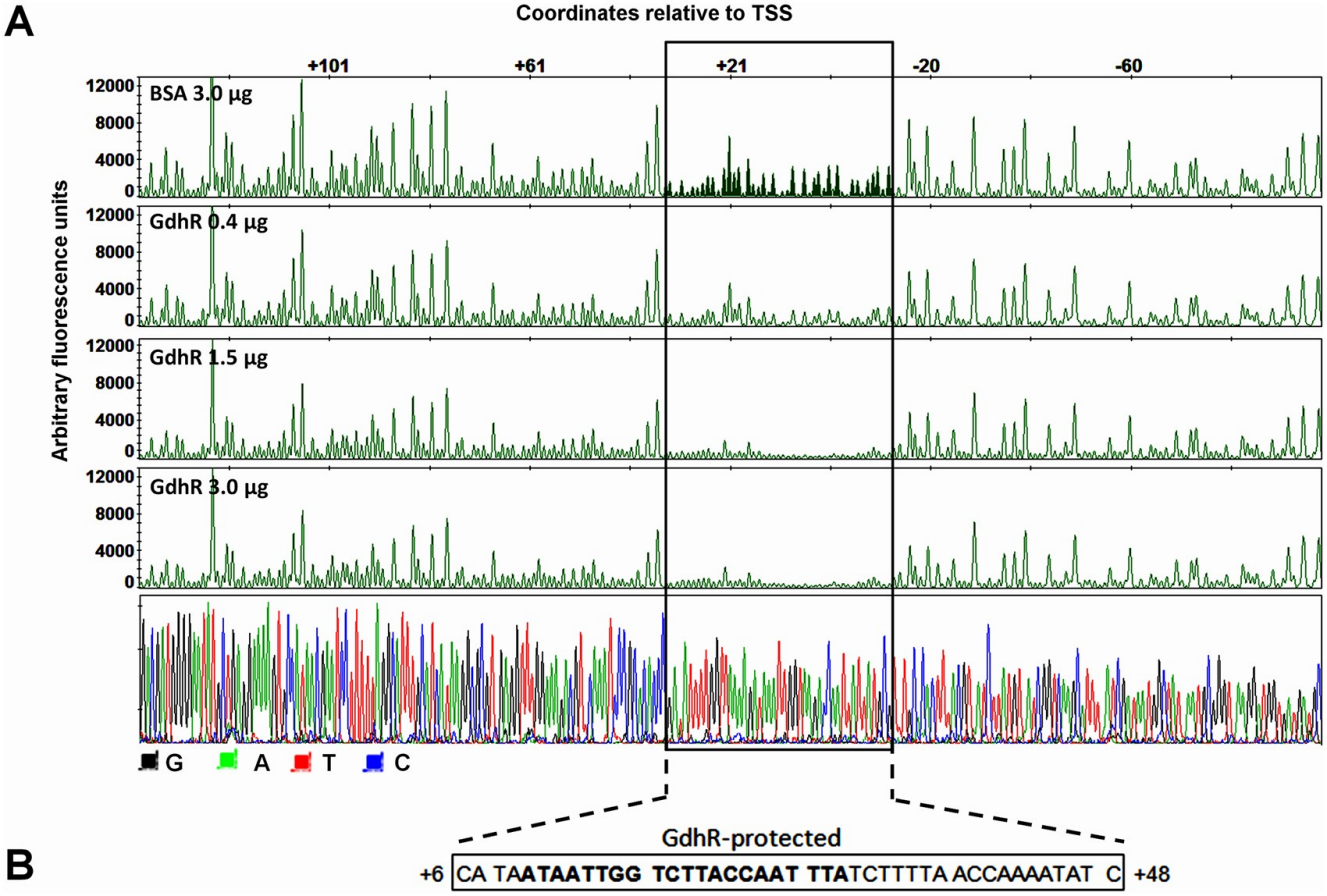
GdhR protects a DNA sequence near the *lctP* promoter. A. A DNA fragment spanning the *lctP* promoter region from nucleotide -192 to +381 (relative to the TSS) was fluorescently-labeled with 6-FAM (coding strand) and HEX (template strand) and incubated with BSA (control reaction) or GdhR prior to digestion with DNase I. The DNase I digestion products were analyzed by capillary electrophoresis. The fluorescence signal corresponding to the HEX probe is shown on the y axis of each electropherogram. Fragment coordinates (relative to the TSS) are shown along the top of the BSA electropherogram. Three electropherograms corresponding to 0.4, 1.5 and 3.0 μg of GdhR reactions are shown. The *lctP* promoter region protected by GdhR is boxed. Dideoxy sequencing reactions were manually-generated using the primer HEX-lctP-DNase and a PCR fragment encoding *lctP* promoter from -192 to +381 (bottom panel). B. DNA sequence of the GdhR-protected region on both strands (the electropherogram corresponding FAM-labeled coding strand is shown in S5 Fig). The consensus DNA-binding motif of the FadR-family of regulators is shown in bold font.

To reconstruct *lctP* transcription *in vitro* we used a PCR-amplified DNA template spanning the *lctP* promoter region, the 5′ untranslated region (5′ UTR) and part of the open reading frame (ORF), and purified *E*. *coli* Sigma-70-saturated RNA polymerase (RNAPσ70). We mapped the *lctP* TSS to a single transcription peak located at an adenine 121 bases upstream the start codon (Fig 4A). A primer extension assay using total RNA isolated *in vivo* from *gdhR* mutant cells revealed a TSS that maps to the same position as with the *in vitro* transcription system (Fig 4A). Since GdhR belongs to the FadR subfamily of the GntR-class of regulators [26] we used the FIMO (Find Individual Motif Occurrences) algorithm [27] and the reported consensus FadR DNA binding motif [28] to generate matches on the *N*. *gonorrhoeae* genome. This analysis revealed an inverted repeat matching the FadR motif located 9 bases downstream of the *lctP* TSS (Fig 4B). DNase I footprinting analysis showed that GdhR protected a single region that included this inverted repeat and extended from +6 to + 48 relative to the *lctP* TSS (Fig 5). The single region of GdhR binding at the *lctP* promoter and the two nucleoprotein complex species observed in the EMSA analysis (Fig 3 lanes d and e) suggest that a change in the oligomerization state of GhdR bound to *lctP* exists at different concentrations of GdhR.

**Fig 6:**
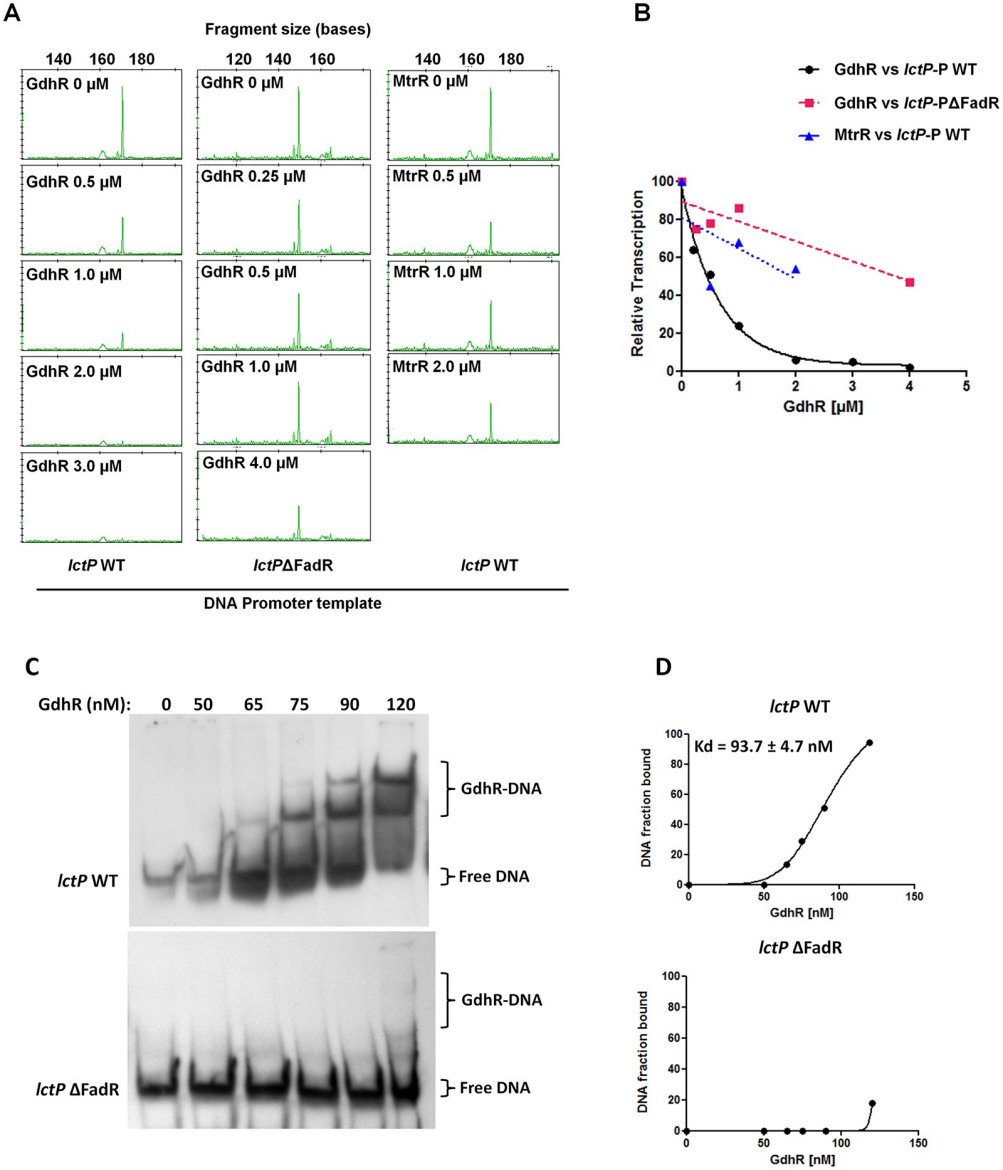
GdhR directly represses *lctP* transcription. A. *In vitro* transcription was conducted in the presence of increasing concentrations of purified GdhR and 50 nM RNAPσ70 using as template a DNA fragment encoding *lctP* promoter from -192 to +381 relative to TSS (*lctP* WT) and the same fragment harboring a 21 bp deletion in the FadR consensus DNA-binding motif (*lctP*ΔFadR). The size of the *lctP* transcript peaks is given at the top x axis. Purified MtrR protein was used as specificity control of the transcription inhibition reaction. B. Transcription inhibition curves were generated by plotting the height of the peaks (considering the 0 μM protein reaction as the 100% transcription point) vs. transcription factor molar concentration. (C) Binding of GdhR to WT *lctP* promoter from -192 to +99 relative to the TSS (*lctP* WT) and to the same promoter fragment harboring the 21 bp deletion in the FadR DNA-binding motif (*lctP*ΔFadR) was compared by EMSA. (D) GdhR binding curves to the WT and mutant *lctP* probes and the dissociation constant (Kd) were determined by densitometry from the EMSA gels adjusting the data to a nonlinear regression analysis using the Hill equation [69].

We used the *in vitro* transcription system to study the effect of GdhR binding to the *lctP* promoter on transcription initiation. Purified GdhR was able to inhibit *lctP* transcription in a concentration-dependent manner with an inhibitory concentration 50% (IC50) of 0.4 μM (0.30–0.52 95% confidence intervals) as determined from the transcription inhibition curve as described in *Materials and Methods* (Fig 6). We used purified MtrR as a specificity control of the assay since it does not directly regulate *lctP* expression [9]. The transcription inhibition curve with MtrR did not show the same exponential decay shape as with GdhR, although it could repress significantly less probably due to nonspecific DNA binding (Fig 6A). To validate the function of the identified GdhR-binding site (Fig 5) on *lctP* transcription regulation, we deleted the 21 bp inverted repeat sequence located within the GdhR protected region and then used this mutant promoter as template for *in vitro* transcription and EMSA. Transcription inhibition (Fig 6A) and binding (Fig 6C) of GdhR to the mutant *lctP* promoter was significantly reduced compared to the WT promoter, which indicates that GdhR requires binding to this inverted repeat sequence in order to repress *lctP* transcription.

### Carbon source regulation of *lctP*

Lactate utilization operons encoding *lctP* and ortholog gene *lldP* can be induced by L-lactate in Gram-negative and -positive bacteria [29–31]. This is achieved by binding of L-lactate to the operon regulator, the GntR-type protein LldR, resulting in the modification of its DNA-binding activity [29–31]. Therefore, we compared the expression of *lctP* in gonococcal cells grown in GC broth supplemented with the standard concentration of D-glucose (22 mM) or with L-lactate (22 mM) (Fig 2). We found that *lctP* expression was enhanced up to 2.5-fold when glucose was replaced by lactate. This effect was, however, GdhR-independent since *gdhR* expression was not affected in the WT strain by the carbon source replacement (S4 Fig) and occurred approximately to same magnitude in the *gdhR* mutant background (Fig 2A). Moreover, we found in our EMSA analysis that neither L-lactate nor glucose impacted the binding of GhdR to the *lctP* promoter (Fig 3, lanes h and i).

**Fig 7:**
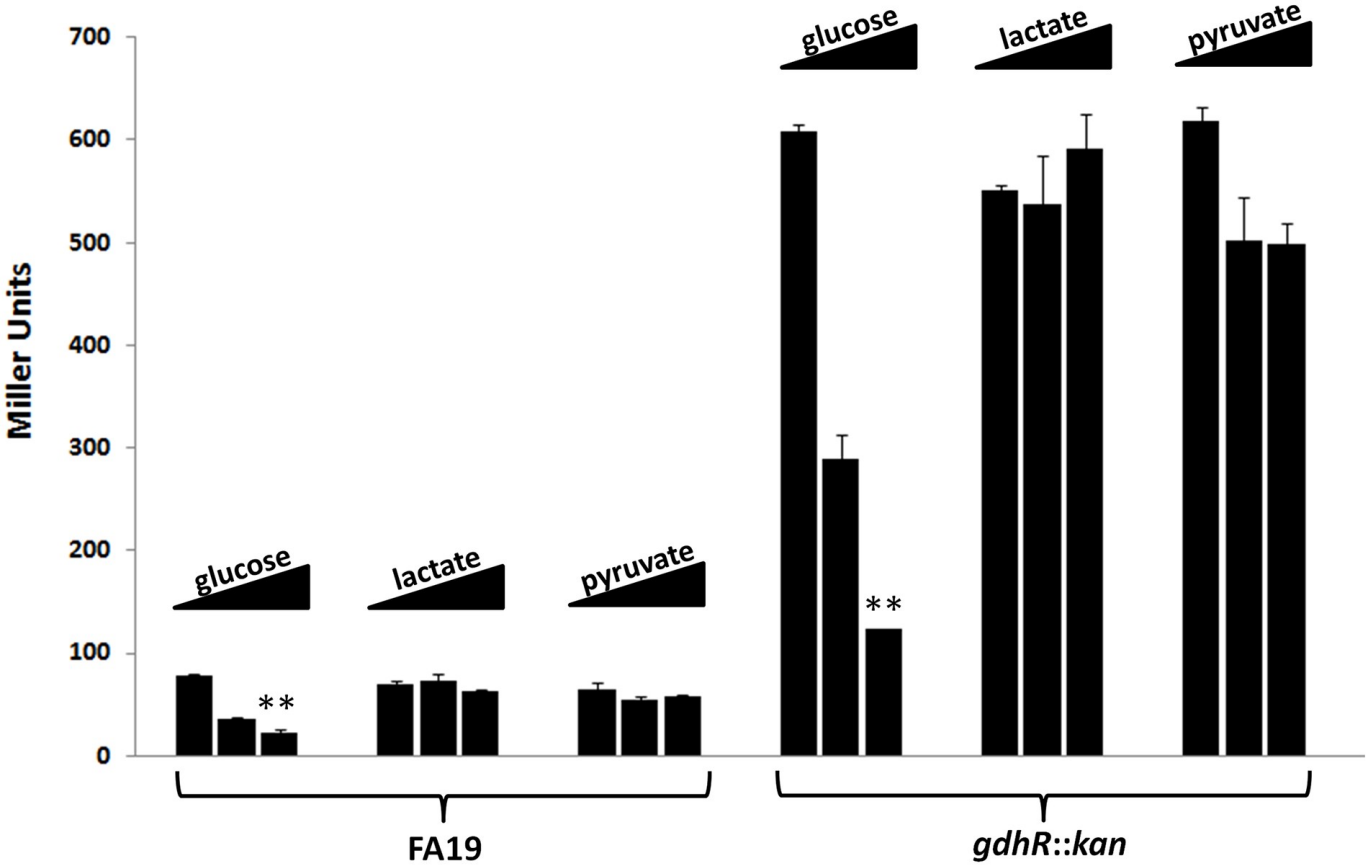
Effect of different carbon sources on *lctP* expression. FA19 reporter strain JC28 and isogenic mutant JC29 (*gdhR*::*kan*) containing an *lctP*-*lacZ* fusion in vector pLES94-*lctP* were grown to stationary phase on GC broth supplemented with a concentration range (1.38, 2.75 and 5.50 mM) for each carbon source. Titration of glucose supplemented cultures was done with a fix concentration of 3 mM L-lactate, and titration with L-lactate or pyruvate was done with a fix concentration of 1.5 mM glucose. β-galactosidase was expressed from the *lctP* transcriptional and translational signals and its activity was determined in Miller units. Data are presented as the mean (bar) plus the standard error of the mean (error bar) of 3 biological samples and two technical replicates each. ** represents significant statistical differences at p<0.01 within each carbohydrate titration group as determined by a non-parametric Kruskal-Wallis test and a Dunn’s posttest.

The above results, however, did not discern between glucose repression and lactate induction of *lctP*. To study the effect of different carbon sources on *lctP* expression we constructed a *lacZ* translational fusion in vector pLES94 in which the *lacZ* gene was fused to the first codon of *lctP* and expressed from the *lctP* transcriptional and translational signals. WT or *gdhR* mutant gonococci bearing the *lctP-lacZ* fusion were grown to stationary phase in GC broth supplemented with either D-glucose, L-lactate or pyruvate in a concentration range of 1 to 6 mM each and β-galactosidase activity was determined as an indicator of LctP levels. This analysis showed that only glucose could repress *lctP* expression at physiological concentration levels, with 55% repression at 2.75 mM and 70% at 5.50 mM relative to 1.38 mM in the WT background (Fig 7, D-glucose titration bars). The glucose repression was again GdhR-independent since it occurred in the *gdhR* mutant background with approximately the same magnitude of effect. This analysis also showed that, different from *E*. *coli* [29], neither lactate nor pyruvate (its immediate oxidation product) can induce *lctP* expression.

### Regulation of *lctP* by GdhR and D-glucose impacts gonococcal resistance to hydrogen peroxide

**Fig 8:**
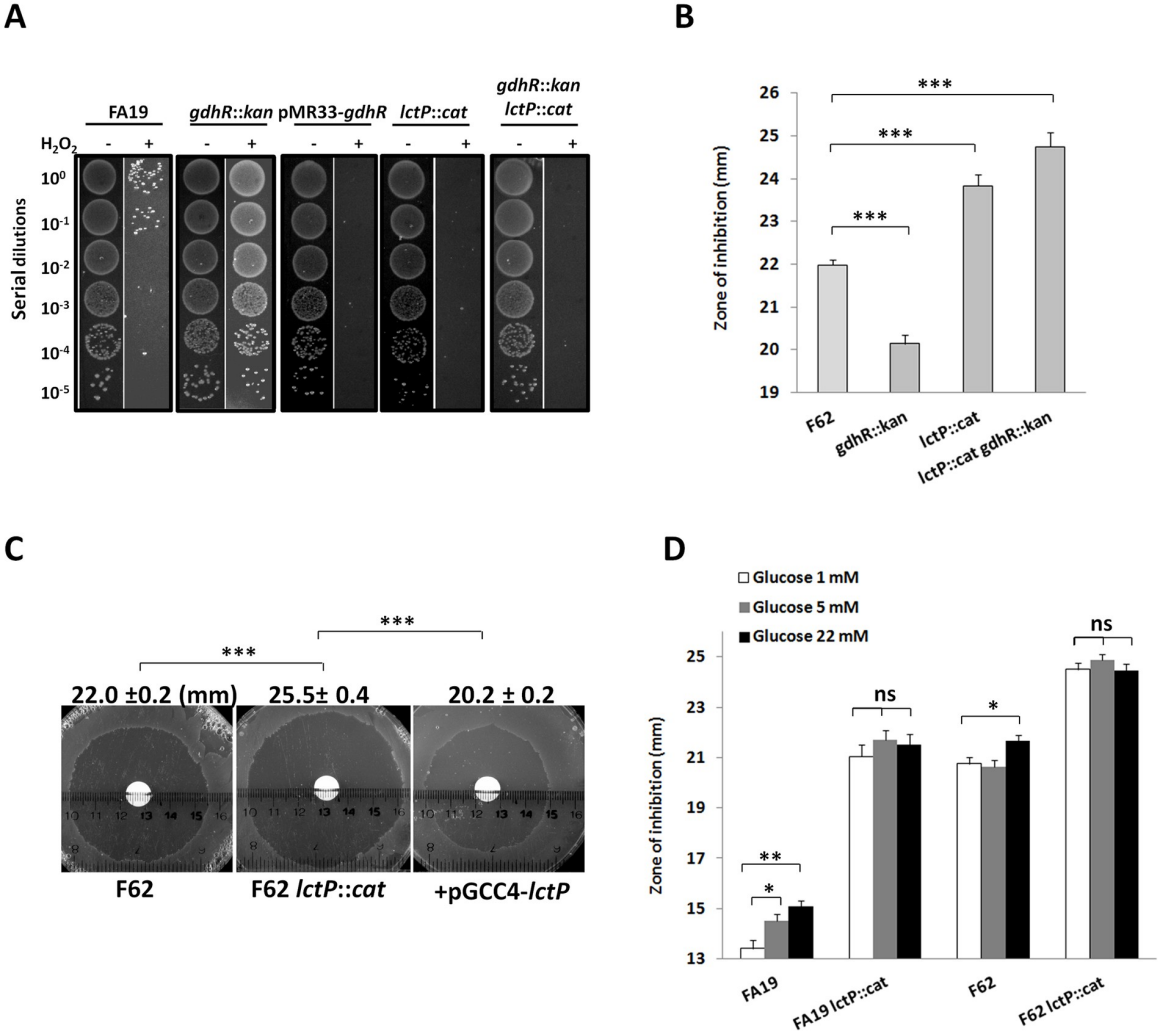
Regulation of *lctP* by GdhR and D-glucose impacts gonococcal resistance to hydrogen peroxide. A. Hydrogen peroxide susceptibility assay in GC-broth. 5·10^7^ CFU/mL of strains FA19 and isogenic mutants *gdhR*::*kan*, *gdhR*-complemented mutant JC02 (pMR33-*gdhR*) in 1 mM IPTG, JC03 (*lctP*::*cat*) and double mutant JC04 (*gdhR*::*kan lctP*::*cat*) were incubated overnight in 9 mM H_2_O_2_ and cell viability was determined by serial dilutions and spot plating as described in Methods. B. The hydrogen peroxide susceptibility of WT strain F62 and its isogenic mutants JC16 (*gdhR*::*kan*), JC05 (*lctP*::*cat*) and JC24 (*gdhR*::*kan lctP*::*cat*) was determined in GC-agar plates using a H_2_O_2_ disk diffusion assay. C. Genetic complementation of the *lctP* mutation. H_2_O_2_ disk diffusion assay was performed on strains F62, GP900 (*lctP*::*cat*) and complemented mutant GP922 (+pGCC4-*lctP*) in 1 mM IPTG (representative picture). D. The hydrogen peroxide susceptibility of WT strains and *lctP* mutant strains JC03 and JC05 of the FA19 and F62 backgrounds respectively was tested in GC-agar plates supplemented with different concentrations of glucose using the H_2_O_2_ disk diffusion assay. Data are represented as the mean (bar) plus the standard error of the mean (error bar) of at least 4 independent measurements for all H_2_O_2_ disk diffusion assays presented. Shown are representative experiments reproduced at least twice. Significant statistical differences were determined using a non-parametric Mann Whitney U-test at p<0.05 (*), 0.01 (**) and 0.001 (***). ns: no significant.

Lactate is one of the few carbon energy sources that can be used by pathogenic *Neisseria*, and phagocyte-derived lactate is available to gonococci and has been reported to enhance the rate of bacterial oxygen metabolism [32]. Exposure of gonococci to superoxide and hydrogen peroxide has been shown to increase bacterial metabolism, specifically L-lactate utilization and lactate dehydrogenase (LDH) activity, [33]. Moreover, expression of *lldD* (NGO0639), encoding a NAD-independent membrane-bound LDH, was enhanced in gonococci exposed to sub-lethal levels of hydrogen peroxide [34]. These results suggest that lactate metabolism is important for resistance to oxidative stress within phagocytes. Therefore, we tested the possibility that *lctP* regulation by GdhR or glucose could impact gonococcal resistance to hydrogen peroxide. For this purpose, we determined the effect of exposure to hydrogen peroxide in GC broth on the survival of WT strain FA19 as well as isogenic single and double *gdhR* or *lctP* mutants. We found that *gdhR* mutants were significantly more resistant to killing by hydrogen peroxide compared to WT cells (Fig 8A). This increased resistance could be reversed by genetic complementation of the *gdhR* allele. In contrast, deletion of *lctP* from the WT strain FA19 made gonococci more susceptible to hydrogen peroxide compared to the WT parent (Fig 8A). Interestingly, deletion of *lctP* in the *gdhR* background rendered the double mutant highly susceptible to hydrogen peroxide, which suggests that the *gdhR* mutation-associated resistant phenotype is epistatic to *lctP*. We also performed a *gdhR-lctP* epistasis analysis in the F62 background, which is the strain used to demonstrate the importance of *lctP* for colonization *in vivo* [19]. Because F62 was significantly more susceptible than FA19 using the hydrogen peroxide susceptibility assay in GC broth (S2 Appendix), we assayed the F62 strains in GC-agar plates using a disk diffusion assay. This analysis confirmed the results shown in the FA19 background (Fig 8B). Further, the hydrogen peroxide susceptibility observed in the *lctP* mutant compared with WT F62 was genetically complemented using previously isolated *lctP* mutant (GP900) and complemented strain expressing *lctP* ectopically from the *lac* promoter (GP922) [19] (Fig 8C).

To test whether glucose regulation of *lctP* impacts the hydrogen peroxide susceptibility phenotype we assayed strains FA19 and F62 and their isogenic *lctP* mutants using the disk diffusion assay in GC-agar plates supplemented with different concentrations of D-glucose (Fig 8D). Glucose in the medium was shown to increase the killing by hydrogen peroxide of WT strains FA19 and F62 but not in the corresponding *lctP* mutants, which shows that the glucose effect depends on the presence of *lctP*.

To test whether the hydrogen peroxide resistant phenotype exhibited by *gdhR* mutant cells could be related to a differential expression of other key genes previously associated with such resistance, we determined their transcript levels in WT FA19 and F62 and their GdhR-negative strains by qRT-PCR (S3 Table). This analysis showed that among *recA* (NGO0741) [35], *mpg* (NGO1686) [36], NGO0554, *katA* (NGO1767) [37], *ccp* (NGO1769) [38], *mntC* (NGO0168) [39] and *msrA* (NGO2059) [40] only *lctP* was differentially regulated by GdhR. In addition, we found that compared to strain FA19, qRT-PCR analysis of RNA extracted from strain F62 showed a significantly lower expression level (3-fold) of *ccp*, which encodes a cytochrome-c peroxidase previously described as an antioxidant-encoding gene [38]. This differential expression could, in part, explain the higher hydrogen peroxide susceptibility of strain F62 compared to FA19.

## Discussion

In this work we uncovered the GdhR regulon in order to follow up previous work that showed that loss of *gdhR* enhanced the fitness of gonococci in a female mouse model of lower genital tract infection [11]. Herein, we showed that GdhR regulates 50 genes, encoding mostly membrane proteins. Interestingly, by comparing the GdhR regulon in gonococci with one reported in the meningococci strain H44/76 [14], we found no overlap between the regulons in these two genetically related *Neisseria*. This confirmed our previous report that despite the high degree of sequence identity in the *gdhR* locus (96%) between these two *Neisseria* species, the regulatory activity of GdhR changes drastically due to differences in promoter sequences targeted by DNA-binding proteins [11].

Within the GdhR regulon we concentrated on *lctP*, which encodes a unique L-lactate transporter and previously reported to be required for effective colonization in the experimental female mouse model of infection [19]. Using protein-DNA binding assays we found that GdhR binds to a 21 base inverted repeat sequence positioned very close and downstream to the *lctP* TSS. This DNA sequence matched the reported consensus DNA-binding motif of FadR-type regulators, which was originally identified by aligning upstream regions corresponding to genes within the regulon of multiple members belonging to this GntR-subfamily [28]. HTH regulators generally bind as dimers to inverted repeat operators [15]. By EMSA, we found evidence for two different GdhR-*lctP* nucleoprotein complexes that coexist and interconvert within a GdhR concentration range. These complexes most likely represent a dimer and tetramer of GdhR bound to DNA since we did not find a second binding site within the *lctP* probe analyzed. Similarly, the lactate utilization operon repressor LldR of *C*. *glutamicum* was found to form two nucleoprotein complexes likely corresponding to dimers and tetramers [30]; nonetheless, the oligomerization state of these regulators has to be further studied. We developed an *in vitro* transcription system using purified components to demonstrate that GdhR binds to its operator sequence to effectively repress *lctP* transcription. Transcription initiation can be inhibited at three different steps: promoter recognition by the RNAP, open complex formation or elongation. From our results it is not clear the precise step in which GdhR represses *lctP* transcription initiation. However, it is likely that GdhR could impose a steric hindrance on the RNAPσ70 recognition of the promoter elements given that RNAPσ70 protects a region from -50 to +20 at σ70 promoters [41,42], a region that overlaps the GdhR operator at *lctP*.

A titration analysis of the *lctP* promoter activity under different concentrations of L-lactate or pyruvate showed that these carbon sources do not have an effect on *lctP* expression within physiological concentration levels. Further, L-lactate did not affect the DNA-binding activity of GdhR. These features deviate significantly from the paradigm in other lactate utilization operons in Gram-negative [29,31] and -positive [30] bacteria, where L,D-lactate can bind the GntR-type LldR repressor/activator to induce transcription of the operon. In *N*. *meningitidis* 2-oxoglutarate can inhibit the binding of GdhR to the promoter of its regulated gene *gdhA* [13]. We found that 2-oxoglutarate did not affect the DNA-binding activity of GdhR at the *lctP* promoter (Fig 3, lane g). We do not discount, however, that an unidentified metabolite can bind to GdhR so as to regulate its binding to the *lctP* promoter. The promoter titration analysis showed that D-glucose in the medium has a repressive effect on *lctP* transcription that is not mediated through GdhR. Carbon catabolite repression (CCR) systems have been largely unexplored in *N*. *gonorrhoeae*. Interestingly, a study to determine the transcriptomic response of *N*. *meningitidis* to glucose in the medium showed that *lctP* is subjected to glucose repression [43]. This group found that HexR, a regulator involved in CCR responses in different proteobacteria [44], controls 28% of the total glucose-regulated genes and *lctP* is not among those. The HexR DNA-binding motif is highly conserved among betaproteobacteria [45]. Using the FIMO algorithm, we scanned the gonococci genome to identify a possible match for the reported meningococcal HexR DNA-binding motif at the *lctP* locus [43]. This analysis revealed that the gonococcal genome possesses all the HexR motifs identified in meningococci but there is not a match in the *lctP* locus (S3 Appendix), which suggests that, equal to meningococci, glucose repression of *lctP* is not mediated by HexR in gonococci. Previously, an ortholog of *ptsK* (NGO0314) was identified among the few phosphotransferase (*pts*) annotated genes in the gonococci genome [46]. PtsK is a serine/threonine protein kinase (HPr(Ser) kinase) that controls CCR responses in G-positive bacteria and a *ptsK* mutant in *B*. *subtilis* is insensitive to transcriptional regulation by CCR [46]. Based on this, we constructed an insertional mutant of *ptsK* in strain FA19 harboring the *lctP-lacZ* fusion (JC41) but found that glucose could still repress *lctP* expression in the absence of the putative PtsK to the same extent as the WT parent (S4 Appendix). Thus, the mechanistic basis for glucose repression of *lctP* remains unknown and will be the subject of further study.

When exposed to sub-lethal concentrations of hydrogen peroxide *N*. *gonorrhoeae* reprograms its transcriptome resulting in phenotypic adaptation to oxidative stress of greater magnitude [33,34]. This adaptation requires new protein synthesis, especially those proteins related to an increase in lactate metabolism such as lactate dehydrogenase [33,34]. In addition, it was shown that gonococci can use phagocyte-derived lactate to enhance its metabolism which stimulates oxygen consumption [32,47]. Thus, gonococci can compete with neutrophils for oxygen resulting in a decreased neutrophil production of reactive oxygen species [48].

Herein, we have shown for the first time that a mutant unable to utilize lactate is more susceptible than its WT parent to killing by hydrogen peroxide. We also showed that regulation of *lctP* by the transcriptional repressor GdhR and by glucose in the medium independently have an impact in the overall resistance of gonococci to hydrogen peroxide. While we do not yet understand why loss of LctP or the presence of glucose increases gonococcal susceptibility to hydrogen peroxide we emphasize that this regulation could be relevant during infection. Thus, gonococci surviving within phagolysosomes will face a glucose-limited environment and may rely on lactate as a carbon source [49] [50]. The level of hydrogen peroxide in the neutrophil phagosome can reach up to 100 mM [51]. To test whether GdhR regulation of *lctP* could be relevant under this hydrogen peroxide levels, we performed the killing assay in GC broth shown in Fig 8A but with a higher gonococci cellular concentration. We found that *gdhR* mutants survived hydrogen peroxide concentrations up to 150 mM. In contrast, the WT strain failed to survive at a concentration of > 50 mM and the complemented mutant, which overrepresses *lctP* (Fig 2), > 13 mM (S6 Fig). To test whether hydrogen peroxide could diminish GdhR binding to the *lctP* promoter, we conducted an EMSA in the presence of hydrogen peroxide with or without ferrous iron that catalyzes the Fenton reaction known to result in highly damaging hydroxyl radicals (S7 Fig). From this analysis we concluded that while hydrogen peroxide has little influence on its own on the GdhR-*lctP* nucleoprotein complex, resulting hydroxyl radicals from the Fenton reaction have a negative impact in the formation of the nucleoprotein complex at lethal hydrogen peroxide concentrations (*i*.*e*. higher than 15 mM and defined as a concentration that kills more than 10% of the cells as per [34] and S6 Fig). This finding and conclusion are consistent with the increased fitness of the *gdhR* mutant in the female mouse model of lower genital tract infection at days 3 and 5 [11], when the influx of polymorphonuclear leukocytes would be elevated compared to earlier stages of infection (A. E. Jerse *et al*., personal communication). This leukocyte influx would potentially increase levels of hydrogen peroxide. Under aerobic conditions, which would facilitate hydrogen peroxide production by leukocytes [52], *lctp* and *lldD* expression is elevated compared to anaerobic conditions [53]. The switch to anaerobic growth has been proposed to occur when gonococci grow within biofilms attached to cervical cells using host-derived nitrite as a terminal electron acceptor [54–56] and is of likely importance when gonococci ascend to the upper female reproductive tract where anaerobes are predominant.

**Fig 9:**
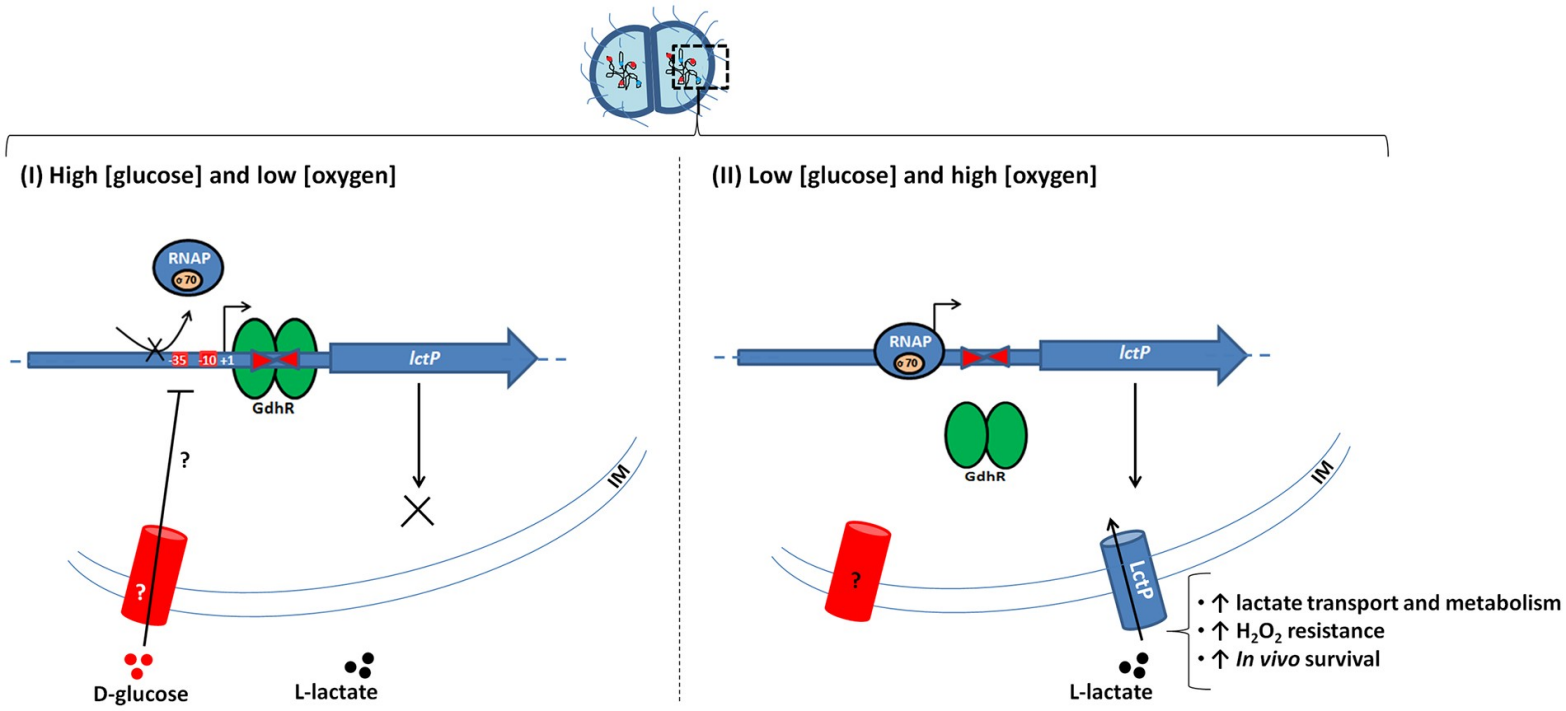
Hypothetical model for the regulation of *N*. *gonorrhoeae lctP* and implications for pathogenesis (based on our data and results published by others, see Discussion). (I) Under conditions of D-glucose availability and low oxygen tension (*i*.*e*. those within a biofilm) the GntR-type regulator GdhR binds to an inverted repeat sequence located 9 bases downstream the TSS (+1). This binding results in transcriptional repression of *lctP* possibly by creating a steric hindrance to the RNA polymerase (RNAP) recognition of the promoter elements (-35 and -10). The presence of D-glucose in the extracellular milieu results in additional repression of *lctP* by an unknown GdhR-independent mechanism. In *N*. *gonorrhoeae* the glucose permease is unknown as wells as any phosphotransferase system involved in carbon catabolite repression. (II) Under conditions of low glucose concentration and oxygen availability (*i*.*e*. those within the neutrophil phagosome) the *lctP* promoter is released from glucose and GdhR repression to reach maximal expression. This increases L-lactate transport and metabolism which results in increased resistance to hydrogen peroxide and survival *in vivo*. Under this condition (oxygen availability or oxidative stress such as hydrogen peroxide treatment), expression of *lldD*, encoding L-lactate dehydrogenase, is also increased.

Taken together, our results and previously published data ([11,19,32–34,47,48,53,55]), we propose a model (Fig 9) in which under conditions of low D-glucose concentration and oxygen availability (*i*.*e*. within neutrophils where oxygen is required for hydrogen peroxide production and glucose is excluded from the phagosome) the *lctP* promoter is released from GdhR and D-glucose repression. This maximally increases LctP levels and therefore lactate transport and metabolism. Lactate dehydrogenase activity is also increased under high oxygen availability and after exposure to hydrogen peroxide. This would allow gonococci to increase their cell population and effectively compete for oxygen availability, which would lower the production of reactive oxygen species by leukocytes and increase the survival of gonococci when faced with oxidative stress. GdhR anti-repression might occur by downregulation of *gdhR* levels through MtrR repression (or possibly other regulators) or by binding of an unidentified metabolite to the ligand-binding domain of GdhR to destabilize its DNA-binding activity. Therefore, the study of the GdhR anti-repression mechanism and the link between D-glucose and repression of *lctP* will help understand how lactate utilization affects the pathogenesis of *N*. *gonorrhoeae* within the human host.

## Materials and methods

### Strains and media

*N*. *gonorrhoeae* strains used in this study are derived from the laboratory strains FA19 and F62 and are described in S4 Table. Gonococcal strains were grown overnight at 37°C under 5% (v/v) CO_2_ on GC agar plates containing Kellogg’s supplements I and II [57]. When indicated glucose in supplement I was replaced by L-lactate or pyruvate at indicated concentrations. Growth in liquid medium was at 37°C with agitation (225 r.p.m.) in GC broth containing Kellogg’s supplements I and II and 0.042% (w/v) sodium bicarbonate. When necessary, culture media were supplemented with ampicillin (Amp; 100 μg/mL), chloramphenicol (Cm; 0.5–1.0 μg/mL), kanamycin (Km; 50 μg/mL), erythromycin (Erm; 1 μg/mL), isopropyl-β-D-thiogalactopyranoside (IPTG; 0.5 to 1.0 mM as indicated) or 5-bromo-4-chloro-3- indolyl-β-D-galactopyranoside (X-gal; 20 μg/mL). *E*. *coli* TOP10 (Life Technologies, Carlsbad, CA) and ER2566 (New England BioLabs, NEB) were used for cloning and protein expression purposes respectively and grown on LB medium.

### Construction of mutant strains and *lctP*-*lacZ* translational reporters

Plasmids and oligonucleotide primers used throughout this work are described in S4 and S5 Tables respectively. To construct complemented strains of mutant FA19 *gdhR*::*kan*, the *gdhR* allele was placed under the *lac* promoter in vectors pGCC4 and pMR33. Briefly, a DNA fragment encoding *gdhR* ORF was amplified by PCR with primers pac1gepR3 and pme1gepR4 from FA19 genomic DNA (gDNA), digested with PacI-PmeI and ligated into similarly digested pGCC4 and pMR33 to create pGCC4-*gdhR* and pMR33-*gdhR* respectively. The inserted *gdhR* allele was confirmed by sequencing with primer pMR33Fw. Vectors pGCC4-*gdhR* and pMR33-*gdhR* (linearized with NheI) were used to transform strain FA19 *gdhR*::*kan* by homologous recombination to generate strains JC01 and JC02 respectively. Transformants were selected on GC agar plates containing Erm. Primer pairs NGO1450-F/pac1gepR3 and pMR33Fw/igaRv were used to confirm by PCR the correct integration of vectors pGCC4-*gdhR* and pMR33-*gdhR* into their respective chromosomal loci. To construct a *gdhR* insertional mutant of strain F62, plasmid pUC18us-*gdhR*::*kan* [11] (linerarized with EcoRI) was used to transform F62 by electroporation using the method described by Dillard J.P. [58] and generating strain JC16. Transformant strains were selected on GC-agar plates containing Km and disruption of the *gdhR* allele was confirmed by PCR with primers gdhR-pTXF and gdhR-pTXR.

To construct *lctP* insertional mutant strains a DNA fragment encoding *lctP* disrupted with a Cm acetyltransferase cassette (*cat*) was amplified by PCR with primers F1-lctP and R1-lctP using gDNA from strain GP900 as a template [19]. The PCR fragment was used to transform FA19, FA19 *gdhR*::*kan*, F62 and JC16 to generate strains JC03, JC04, JC05 and JC24 respectively. Transformant strains were selected on GC-agar plates containing Cm and disruption of the *lctP* allele was confirmed by PCR with primers lctP-check and lctP-R2.

To create *lctP* reporter strains a transcriptional/translational *lctP*-*lacZ* fusion was created in vector pLES94 [59]. Briefly, a DNA fragment encompassing the *lctP* promoter, the 5’ UTR and the first codon was amplified by PCR using primers lctPlacZ-F and lctPlacZ-R and FA19 gDNA. The resulting PCR fragment was ligated into BamHI-digested pLES94 to create pLES94-*lctP*. Vector pLES94-*lctP* was linearized with HindIII and used to transform FA19 and FA19 *gdhR*::*kan* to generate strains JC28 and JC29 respectively. Transformants were selected on GC-agar plates containing Cm and the integration of the *lacZ* fusion at the *proAB* locus was confirmed by PCR with primers proABFw and lacZRv.

### Extraction of total RNA and qRT-PCR

*N*. *gonorrhoeae* cultures were grown in GC broth at 37°C with agitation to late exponential phase before being used for RNA purification. One mL samples were centrifuged, resuspended in 200 μL RNA*later* solution (Ambion) and incubated 10 min on ice. Total RNA extraction was conducted using the RNeasy mini kit (Qiagen) following the manufacturer’s protocol. Contamination with gDNA was removed using the Turbo DNA-free Kit (Invitrogen). For qRT-PCR the DNase I-digested total RNA samples were reverse transcribed using the QuantiTect Reverse Transcription Kit (QIAGEN). For primer extension assay and RNA-Seq, DNase I-digested total RNA samples were further concentrated and cleaned-up using the RNeasy MinElute Cleanup Kit (QIAGEN). The integrity of the purified RNA samples was determined by formaldehyde agarose gel electrophoresis as described before [60].

qRT-PCR was conducted using the IQ SYBR Green Supermix and a CFX Connect Real Time System (Bio-Rad Laboratories). Relative expression values were calculated as 2^(CT reference − *CT* target)^, where CT is the fractional threshold cycle. The level of *recA* mRNA and 16S rRNA were used as internal reference. The following primer pairs were used to quantify relative mRNA levels: recAqFw/recAqRv for *recA*, 16Smai-RTF/16Smai-RTR for 16S rRNA, lctPqFw/lctPqRv for *lctP*, gepR_qRT_F/gdhR_qRT_R2 for *gdhR*, rmpM_qRT_F/rmpM_qRT_R for *rmpM*, mtrC_qRT_F/mtrC_qRT_R for *mtrC*, mtrR_qRT_F/mtrR_qRT_R for *mtrR*, 1686qRT-F/1686qRT-T for *mpg*, 0554qRT-F/0554qRT-R for NGO0554, katAqRT-F/katAqRT-R for *katA*, ccp_qRT_F1/ccp_qRT_R1 for *ccp*, mntCqRT-F/mntCqRT-R for *mntC*, and msrAqRT-F/msrAqRT-R for *msrA*.

### RNA-Seq and bioinformatics analysis

RNA-Sequencing was performed on the Illumina NextSeq 500 as described by the manufacturer (Illumina Inc., San Diego, CA). Briefly, the quality of the total RNA was assessed using the Agilent 2100 Bioanalyzer. RNA with a RNA Integrity Number (RIN) of 7.0 or above was used for sequencing library preparation. Library preparation was done using the Agilent SureSelect Strand Specific mRNA library kit as per the manufacturer’s instructions (Agilent, Santa Clara, CA). Library construction began with ribosome reduction using the RiboMinus rRNA depletion kit for bacteria (Invitrogen). The resulting RNA was randomly fragmented with cations and heat, which was followed by first strand synthesis using random primers with inclusion of Actinomycin D (2.4ng/μL final concentration). Second strand cDNA production was done with standard techniques, the ends of the resulting cDNA were made blunt, A-tailed and adaptors ligated for amplification and indexing to allow for multiplexing during sequencing. The cDNA libraries were quantitated using qPCR in a Roche LightCycler 480 with the Kapa Biosystems kit for Illumina library quantitation (Kapa Biosystems, Woburn, MA) prior to cluster generation. Cluster generation was performed according to the manufacturer’s recommendations for onboard clustering (Illumina).

For the RNA-Seq bioinformatics analysis, softwares deposited in the public ABIMS Galaxy tool shed (Station Biologique de Roscoff-CNRS-Sorbonne University) were used. Briefly, paired-end fastq files generated by the sequencing platform were aligned to the *N*. *gonorrhoeae* FA19 genome (GenBank assembly accession: GCA_000273665.1) using the Tophat2 algorithm to generate BAM files. Transcript differential expression between samples (n = 2) was determined using the aligned BAM files and the Cuffdiff algorithm. Finally, the BAM files, the Tophat2 alignment rates and the RNA-Seq laboratory and bioinformatics methods were deposited in the Gene Expression Omnibus (GEO) [61] with GEO series accession number GSE134959.

### Protein purification

MtrR was purified as described before [62]. To purify GdhR, the gene was amplified with primers gdhR-pTXF and gdhR-pTXR from FA19 gDNA and cloned into NdeI/SapI-digested pTXB1 (NEB) to generate pTXB1-*gdhR*. The GdhR-intein-CBD encoding fusion in pTXB1-*gdhR* was confirmed by DNA sequencing using T7 universal and Mxe Intein II reverse primers (NEB). GdhR encoding gene was expressed from the T7 promoter and the protein purified from *E*. *coli* French press-generated lysates using the NEB IMPACT protein purification system following the company protocol.

### Electrophoresis mobility shift assay (EMSA)

EMSAs were conducted using the second-generation digoxigenin (DIG) gel shift kit (Roche Applied Sciences, Madison, WI) as previously described [63]. Briefly, 12 fmol of DIG-labeled DNA fragments were incubated with increasing concentrations of purified GdhR protein for 25 min at 30°C before being separated by electrophoresis in 5% Mini-Protean TBE Precast Gels (Bio-Rad) and transferred to nylon membranes. The protein-DNA binding reactions were carried out in 20 μL of 20 mM Hepes, pH 7.6, 1 mM EDTA, 10 mM (NH_4_)_2_SO_4_, 1 mM DTT, 0.2% (v/v) Tween-20, 30 mM KCl and 1.25 ng/μL Type XV calf thymus DNA. Gel images were developed using an anti-DIG Fab fragment-AP conjugate and chemiluminescence detection, acquired with the Gel Doc XR Molecular Imager (Bio-Rad) and processed with the Image Lab software (Bio-Rad). Specificity of GdhR binding to the DIG-labeled *lctP* promoter was tested by adding to the binding reactions a 100-fold excess of unlabeled DNA fragments encoding the house-keeping gene *recA* promoter region or 16S ribosomal RNA. DNA fragments were generated by PCR from *N*. *gonorrhoeae* FA19 gDNA with primer pairs GdhR-EMSA-F/GdhR-EMSA-R for *lctP* promoter, recAP-F/recAP-R for *recA* promoter and 16Smai-RTF/16Smai-RTR for 16S ribosomal RNA.

### *In vitro* transcription and primer extension reactions

*In vitro* transcription assays were carried out as described before [64]. Briefly, a DNA fragment encoding *lctP* promoter, 5’ UTR and part of the coding sequence was amplified from FA19 gDNA with primers GdhR-EMSA-F and lctPqRv and used as a template. One μg of template DNA was incubated with either purified GdhR or MtrR proteins before being transcribed with 1.0 unit of *E*. *coli* RNAPσ70 (NEB) and nucleotide triphosphates. The transcription reactions were digested with 3 units of RQ1 DNase I (Promega). The RNA transcripts were purified with the QIAGEN RNeasy MinElute cleanup kit before being reverse-transcribed using HEX-labeled primer HEX-lctP-IvT (complementary to the *lctP* ORF) and the SuperScript II Reverse Transcriptase system (Invitrogen) following the company protocol. A 369 bp HEX-labeled DNA standard generated with primers GdhR-EMSA-F and HEX-lctP-IvT was added to each sample to a final concentration of 0.75 ng/μL. For primer extension assay 16.4 μg of total RNA were isolated from strain FA19 *gdhR*::*kan* as described in the above section (*Extraction of total RNA*), annealed with primer HEX-lctP-IvT and extended similarly to the *in vitro* transcription RNA products.

To create a mutant *lctP* promoter template lacking the identified FadR DNA binding motif (*lctP*ΔFadR), overlap extension PCRs were carried out with primer pairs GdhR-EMSA-F/MotifDel-R and MotifDel-F/lctPqRv. Primers MotifDel-R and MotifDel-F are complementary to one another, for which in the second-round of PCR, aliquots of both first-round PCRs were mixed and used as template with primers GdhR-EMSA-F and lctPqRv to generate *lctP*ΔFadR.

### Quantitative analysis of *in vitro* transcription and primer extension reactions

The resulting fluorescently-labeled cDNA fragments were analyzed in a 3730 capillary sequencer (Applied Biosystems) and the sequencing files were visualized with the GeneMapper software v.4.0 (Applied Biosystems). To accurately assign a nucleotide base to *in vitro* transcription or primer extension peaks, a sequencing ladder was generated using the above *lctP* template DNA, primer HEX-lctP-IvT and the Thermo Sequenase Dye Primer Manual cycle sequencing kit (USB Corporation) as described before [65]. The GeneMapper software was used to generate alignments between the electropherograms of the transcription reactions and dideoxy sequencing reactions to determine the size and start nucleotide of the *lctP* transcripts. The effect of GdhR and MtrR on *lctP in vitro* transcription was estimated from the height of *lctP* transcript peaks present in the electropherograms. Transcription inhibition curves were generated with the aid of GraphPad Prism 5.0 (GraphPad, San Diego, CA) using the log (inhibitor) vs. response-variable slope nonlinear regression analysis.

### DNase I footprinting

A PCR fragment spanning the *lctP* promoter was amplified using the 6-carboxyfluorescein (FAM)- and 6-carboxy- 2’,4,4’,5’,7,7’- hexachlorofluorescein (HEX)-labeled primers FAM-lctP-DNase and HEX-lctP-DNase. GdhR protein binding to the labeled DNA probe and DNase I digestion reactions were performed as described previously [65]. Detection of the DNase I digestion peaks was carried out in a 3730 capillary sequencer (Applied Biosystems) and the alignment of the corresponding electropherograms was generated using GeneMapper software v.4.0 (Applied Biosystems). Negative control reactions were done using BSA at the same mass concentration used for GdhR. A PCR DNA template was amplified with primers GdhR-EMSA-F and lctPqRv to generate a sequence ladder for each strand with primers FAM-lctP-DNase (coding strand) and HEX-lctP-DNase (template strand) as described for the *In vitro transcription* section and as previously described [65].

### β-galactosidase activity

β-galactosidase enzymatic activity was determined using the substrate o-nitro phenyl-β-D-galactopyranoside (ONPG) as described by Miller J.H. before [66]. β-galactosidase activities are given in Miller units using the formula [1,000 × OD_420nm_ / (*t* × *v* × OD_600nm_)], where *t* is the reaction time in min and *v* is the volume of cell lysates in mL per reaction.

### Hydrogen peroxide susceptibility assays

H_2_O_2_ susceptibility assays were performed in liquid medium with gonococcal cells collected from overnight growth on GC-agar plates. The bacteria were then resuspended to 5·10^7^ CFU/mL in 200 μL of GC liquid medium containing Kellogg’s supplement I and II, 0.042% NaHCO_3_, 3 mM L-lactate and 9 mM H_2_O_2_. After overnight incubation at 37°C cell viability was assessed by dilution in GC broth and spot plating on GC-agar plates. To test susceptibility of gonococci to different H_2_O_2_ concentrations a variation of the liquid assay was made consisting of overnight incubation of 5·10^8^ CFU/mL gonococcal cells in 96-well flat-bottom sterile plates with lid at 37°C under 5% (v/v) CO_2_. Cell viability was assessed this time with the AlamarBlue dye (Bio-Rad) and fluorescence reading at 560/590 nm (excitation/emission), considering 100% survival the cellular growth in wells without H_2_O_2_. For the hydrogen peroxide susceptibility using the disk diffusion assay, a 10^9^ CFU/mL suspension of gonococcal cells was spread onto GC-agar plates containing Kellogg’s supplement I and II and 3 mM L-lactate and incubated 30 min at 37°C under 5% (v/l) CO_2_. Then, Whatman filter disks (1 cm) presoaked in 3% H_2_O_2_ were placed on top of the plates and further incubated overnight before growth inhibition zones were measured in mm from the edge of the disk.

## Supporting information

S1 Fig Kinetics of expression of *gdhR* in gonococcal cells. Relative levels of *gdhR*, *mtrR*, *mtrC* and *rmpM* mRNA were determined by qRT-PCR using *recA* (A) and 16S rRNA (B) as internal reference genes. Total RNA samples were collected from WT strain FA19 at different optical density (OD) points of its growth in GC broth. Data are presented as the mean (bar) plus the standard deviation (error bar) of 3 biological samples. * represents significant statistical differences at p<0.05 as determined by a non-parametric Kruskal-Wallis test and Dunn posttest.
(TIF)

S2 Fig GdhR repression of *lctP* in the F62 background. Relative levels of *lctP* mRNA were determined by qRT-PCR using *recA* (A) and 16S rRNA (B) as internal reference genes. Total RNA samples were collected from WT strain F62 and its isogenic mutant JC16 (*gdhR*::*kan*) grown in GC broth to late-logarithmic phase. Data are presented as the mean (bar) plus the standard deviation (error bar) of 3 biological samples. Significant statistical differences (p<0.01) were determined by a T-test.
(TIF)

S3 Fig Alignment of the *lpxH-lctP* intergenic region among different *N*. *gonorrhoeae* strains. The promoter elements (-35 and -10), the TSS (+1) and Shine-Delgarno (SD) regions are underlined. A G-C polymorphism is highlighted in yellow. The end and start of ORFs corresponding to *lpxH* and *lctP* are indicated under the sequence.
(TIF)

S4 Fig Expression profile of *gdhR* under growth on different carbon sources. Relative levels of *gdhR* mRNA were determined by qRT-PCR using *recA* (A) and 16S rRNA (B) as internal reference genes. Total RNA samples were collected from WT strain FA19 grown to late-logarithmic phase in GC broth supplemented either with D-glucose (22 mM) or L-lactate (22 mM). Data are presented as the mean (bar) plus the standard deviation (error bar) of 4 biological samples.
(TIF)

S5 Fig GdhR DNase I footprint of the *lctP* promoter coding strand. A DNA fragment spanning the *lctP* promoter region from nucleotide -192 to +381 (relative to the TSS) was fluorescently-labeled with 6-FAM (coding strand) and HEX (template strand) and incubated with BSA (control reaction) or GdhR prior to digestion with DNase I. The DNase I digestion products were analyzed by capillary electrophoresis. The fluorescence signal corresponding to the 6-FAM probe is shown on the y axis of each electropherogram. Fragment coordinates (relative to the TSS) are shown along the top of the BSA electropherogram. Three electropherograms corresponding to 0.4, 3.0 and 6.0 μg of GdhR reactions are shown. The *lctP* promoter region protected by GdhR is boxed. Dideoxy sequencing reactions were manually-generated using the primer FAM-lctP-DNase and a PCR fragment encoding *lctP* promoter from -192 to +381 (bottom panel).
(TIF)

S6 Fig Mutants lacking *gdhR* are highly resistant to hydrogen peroxide. (A) Gonococcal cells (5·10^8^ CFU/mL) of the WT FA19 strain, its isogenic mutant *gdhR*::*kan* and *gdhR*-complemented mutant JC02 (pMR33-*gdhR* + 1 mM IPTG) were exposed to different concentration of hydrogen peroxide in GC broth and grown overnight in 96-wells plates. Cell viability was determined with the Alamar blue dye. (B) Graphical representation of killing by H_2_O_2_ from the fluorescence reading data considering the 0 mM point as 100% growth. Representative experiment of at least two.
(TIF)

S7 Fig Effect of hydrogen peroxide and ferrous iron on the GdhR-*lctP* nucleoprotein complex formation. (A) Shown are results from an EMSA experiment that used purified GdhR (120 nanomolar) and a digoxigenin-labeled DNA encompassing the *lctP* promoter (−313 to −23 relative to the start codon). Binding reactions were performed in the presence of increasing concentrations of either H_2_O_2_ alone or H_2_O_2_ and 12 μM FeSO_4_ (Fe^2+^) that catalyzes the Fenton reaction. The mobility of free DNA and of the nucleoprotein complexes are indicated at the right of the gel. (B) The effect of H_2_O_2_ and Fe^2+^ on the top nucleoprotein complex formation (red arrow) was examined by densitometry of the EMSA gel using the ImageLab 6.0 software.
(TIF)

S1 Table Differential gene expression between WT and *gdhR* mutant. Excel file containing the list of GdhR-regulated genes generated by a transcript differential expression analysis of the WT vs. *gdhR*::*kan* mutant sequencing files. Tab-1 shows differentially expressed genes. Tab-2 shows differentially expressed genes at a fold-change >2. Tab-3 shows the expression levels (in FPKM) of all sequenced genes.
(XLSX)

S2 Table Differential gene expression between *gdhR* mutant and complemented strains. Excel file containing the list of GdhR-regulated genes generated by a transcript differential expression analysis of the *gdhR*::*kan* mutant vs. pGCC4-*gdhR* complemented mutant (JC01) sequencing files. Tab-1 shows differentially expressed genes. Tab-2 shows differentially expressed genes at a fold-change >2. Tab-3 shows the expression levels (in FPKM) of all sequenced genes.
(XLSX)

S3 Table Relative expression of genes required for the hydrogen peroxide oxidative damage response in *N*. *gonorrhoeae*. (DOCX)

S4 Table Strains and plasmids used in this study. (DOCX)

S5 Table Oligonucleotide primers used in this study. (DOCX)

S1 Appendix Alignment of the *gdhR* allele and upstream sequence from different *N*. *gonorrhoeae* strains. (DOCX)

S2 Appendix Sensitivity of *N*. *gonorrhoeae* strains to hydrogen peroxide in GC broth. (DOCX)

S3 Appendix Bioinformatic detection of the *N*. *meningitidis* HexR DNA-binding motif within the *N*. *gonorrhoeae* FA1090 genome using the FIMO algorithm. (DOCX)

S4 Appendix Effect of the *ptsK* allele deletion on the glucose-mediated repression of *lctP*. (DOCX)

## References

[1] Reported STDs in the United States, 2016 High Burden of STDs Threaten Millions of Americans In: Prevention CfDCa, editor. CDC Fact Sheet: Center for Disease Control and Prevention.

[2] Jane Rowley SVH, Eline Korenromp, Nicola Low, Magnus Unemo, Laith J Abu-Raddad, R Matthew Chico, Alex Smolak, Lori Newman, Sami Gottlieb, Soe Thwin, Nathalie Brouteta and Melanie M Taylor (2019) Chlamydia, gonorrhoea, trichomoniasis and syphilis: global prevalence and incidence estimates, 2016. Bulletin of the World Health Organization: 1–43. 10.2471/BLT.18.228486 PMC6653813 31384073

[3] National Center for HIV/AIDS, Viral Hepatitis, STD, and TB Prevention. Center for Disease Control and Prevention. Atlanta, GA. Press release: New Warning Signs that Gonorrhea Treatment May be Losing Effectiveness. 2016 Sep 21. https://www.cdc.gov/nchhstp/newsroom/2016/2016-std-prevention-conference-press-release.html

[4] Zhao S, Duncan M, Tomberg J, Davies C, Unemo M, et al (2009) Genetics of chromosomally mediated intermediate resistance to ceftriaxone and cefixime in *Neisseria gonorrhoeae*. Antimicrob Agents Chemother 53: 3744–3751. 10.1128/AAC.00304-09 19528266 PMC2737842

[5] Ameyama S, Onodera S, Takahata M, Minami S, Maki N, et al (2002) Mosaic-like structure of penicillin-binding protein 2 Gene (penA) in clinical isolates of *Neisseria gonorrhoeae* with reduced susceptibility to cefixime. Antimicrob Agents Chemother 46: 3744–3749. 10.1128/AAC.46.12.3744-3749.2002 12435671 PMC132769

[6] Osaka K, Takakura T, Narukawa K, Takahata M, Endo K, et al (2008) Analysis of amino acid sequences of penicillin-binding protein 2 in clinical isolates of *Neisseria gonorrhoeae* with reduced susceptibility to cefixime and ceftriaxone. J Infect Chemother 14: 195–203. 10.1007/s10156-008-0610-7 18574654

[7] Olesky M, Hobbs M, Nicholas RA (2002) Identification and analysis of amino acid mutations in porin IB that mediate intermediate-level resistance to penicillin and tetracycline in *Neisseria gonorrhoeae*. Antimicrob Agents Chemother 46: 2811–2820. 10.1128/AAC.46.9.2811-2820.2002 12183233 PMC127413

[8] Hagman KE, Pan W, Spratt BG, Balthazar JT, Judd RC, et al (1995) Resistance of *Neisseria gonorrhoeae* to antimicrobial hydrophobic agents is modulated by the mtrRCDE efflux system. Microbiology 141 (Pt 3): 611–622. 7711899 10.1099/13500872-141-3-611

[9] Folster JP, Johnson PJ, Jackson L, Dhulipali V, Dyer DW, et al (2009) MtrR modulates rpoH expression and levels of antimicrobial resistance in *Neisseria gonorrhoeae*. J Bacteriol 191: 287–297. 10.1128/JB.01165-08 18978065 PMC2612434

[10] Folster JP, Shafer WM (2005) Regulation of mtrF expression in *Neisseria gonorrhoeae* and its role in high-level antimicrobial resistance. J Bacteriol 187: 3713–3720. 10.1128/JB.187.11.3713-3720.2005 15901695 PMC1112036

[11] Rouquette-Loughlin CE, Zalucki YM, Dhulipala VL, Balthazar JT, Doyle RG, et al (2017) Control of gdhR Expression in *Neisseria gonorrhoeae* via Autoregulation and a Master Repressor (MtrR) of a Drug Efflux Pump Operon. MBio 8. 10.1128/mBio.00449-17 PMC5388806 28400529

[12] Claus H, Maiden MC, Wilson DJ, McCarthy ND, Jolley KA, et al (2005) Genetic analysis of meningococci carried by children and young adults. J Infect Dis 191: 1263–1271. 10.1086/428590 15776372

[13] Pagliarulo C, Salvatore P, De Vitis LR, Colicchio R, Monaco C, et al (2004) Regulation and differential expression of gdhA encoding NADP-specific glutamate dehydrogenase in *Neisseria meningitidis* clinical isolates. Mol Microbiol 51: 1757–1772. 10.1111/j.1365-2958.2003.03947.x 15009900

[14] Monaco C, Tala A, Spinosa MR, Progida C, De Nitto E, et al (2006) Identification of a meningococcal L-glutamate ABC transporter operon essential for growth in low-sodium environments. Infect Immun 74: 1725–1740. 10.1128/IAI.74.3.1725-1740.2006 16495545 PMC1418650

[15] Rigali S, Derouaux A, Giannotta F, Dusart J (2002) Subdivision of the helix-turn-helix GntR family of bacterial regulators in the FadR, HutC, MocR, and YtrA subfamilies. J Biol Chem 277: 12507–12515. 10.1074/jbc.M110968200 11756427

[16] Aravind L, Anantharaman V (2003) HutC/FarR-like bacterial transcription factors of the GntR family contain a small molecule-binding domain of the chorismate lyase fold. FEMS Microbiol Lett 222: 17–23. 10.1016/S0378-1097(03)00242-8 12757941

[17] Zheng M, Cooper DR, Grossoehme NE, Yu M, Hung LW, et al (2009) Structure of *Thermotoga maritima* TM0439: implications for the mechanism of bacterial GntR transcription regulators with Zn2+-binding FCD domains. Acta Crystallogr D Biol Crystallogr 65: 356–365. 10.1107/S0907444909004727 19307717 PMC2659884

[18] Rigali S, Schlicht M, Hoskisson P, Nothaft H, Merzbacher M, et al (2004) Extending the classification of bacterial transcription factors beyond the helix-turn-helix motif as an alternative approach to discover new cis/trans relationships. Nucleic Acids Res 32: 3418–3426. 10.1093/nar/gkh673 15247334 PMC443547

[19] Exley RM, Wu H, Shaw J, Schneider MC, Smith H, et al (2007) Lactate acquisition promotes successful colonization of the murine genital tract by *Neisseria gonorrhoeae*. Infect Immun 75: 1318–1324. 10.1128/IAI.01530-06 17158905 PMC1828543

[20] Parsons NJ, Boons GJ, Ashton PR, Redfern PD, Quirk P, et al (1996) Lactic acid is the factor in blood cell extracts which enhances the ability of CMP-NANA to sialylate gonococcal lipopolysaccharide and induce serum resistance. Microb Pathog 20: 87–100. 10.1006/mpat.1996.0008 8722097

[21] Yates E, Gao L, Woodcock N, Parsons N, Cole J, et al (2000) In a medium containing glucose, lactate carbon is incorporated by gonococci predominantly into fatty acids and glucose carbon incorporation is increased: implications regarding lactate stimulation of metabolism. Int J Med Microbiol 290: 627–639. 10.1016/S1438-4221(00)80012-0 11200544

[22] Atack JM, Ibranovic I, Ong CL, Djoko KY, Chen NH, et al (2014) A role for lactate dehydrogenases in the survival of *Neisseria gonorrhoeae* in human polymorphonuclear leukocytes and cervical epithelial cells. J Infect Dis 210: 1311–1318. 10.1093/infdis/jiu230 24737798 PMC4215069

[23] Blake MS, Wetzler LM, Gotschlich EC, Rice PA (1989) Protein III: structure, function, and genetics. Clin Microbiol Rev 2 Suppl: S60–63. 2497964 10.1128/cmr.2.suppl.s60 PMC358079

[24] Smith H, Yates EA, Cole JA, Parsons NJ (2001) Lactate stimulation of gonococcal metabolism in media containing glucose: mechanism, impact on pathogenicity, and wider implications for other pathogens. Infect Immun 69: 6565–6572. 10.1128/IAI.69.11.6565-6572.2001 11598023 PMC100028

[25] Smith H, Tang CM, Exley RM (2007) Effect of host lactate on gonococci and meningococci: new concepts on the role of metabolites in pathogenicity. Infect Immun 75: 4190–4198. 10.1128/IAI.00117-07 17562766 PMC1951187

[26] Haydon DJ, Guest JR (1991) A new family of bacterial regulatory proteins. FEMS Microbiol Lett 63: 291–295. 10.1016/0378-1097(91)90101-f 2060763

[27] Grant CE, Bailey TL, Noble WS (2011) FIMO: scanning for occurrences of a given motif. Bioinformatics 27: 1017–1018. 10.1093/bioinformatics/btr064 21330290 PMC3065696

[28] Suvorova IA, Korostelev YD, Gelfand MS (2015) GntR Family of Bacterial Transcription Factors and Their DNA Binding Motifs: Structure, Positioning and Co-Evolution. PLoS One 10: e0132618 10.1371/journal.pone.0132618 26151451 PMC4494728

[29] Aguilera L, Campos E, Gimenez R, Badia J, Aguilar J, et al (2008) Dual role of LldR in regulation of the lldPRD operon, involved in L-lactate metabolism in *Escherichia coli*. J Bacteriol 190: 2997–3005. 10.1128/JB.02013-07 18263722 PMC2293229

[30] Georgi T, Engels V, Wendisch VF (2008) Regulation of L-lactate utilization by the FadR-type regulator LldR of *Corynebacterium glutamicum*. J Bacteriol 190: 963–971. 10.1128/JB.01147-07 18039772 PMC2223578

[31] Gao C, Hu C, Zheng Z, Ma C, Jiang T, et al (2012) Lactate utilization is regulated by the FadR-type regulator LldR in *Pseudomonas aeruginosa*. J Bacteriol 194: 2687–2692. 10.1128/JB.06579-11 22408166 PMC3347178

[32] Britigan BE, Klapper D, Svendsen T, Cohen MS (1988) Phagocyte-derived lactate stimulates oxygen consumption by *Neisseria gonorrhoeae*. An unrecognized aspect of the oxygen metabolism of phagocytosis. J Clin Invest 81: 318–324. 10.1172/JCI113323 3123517 PMC329573

[33] Fu HS, Hassett DJ, Cohen MS (1989) Oxidant stress in *Neisseria gonorrhoeae*: adaptation and effects on L-(+)-lactate dehydrogenase activity. Infect Immun 57: 2173–2178. 2543633 10.1128/iai.57.7.2173-2178.1989 PMC313857

[34] Quillin SJ, Hockenberry AJ, Jewett MC, Seifert HS (2018) *Neisseria gonorrhoeae* Exposed to Sublethal Levels of Hydrogen Peroxide Mounts a Complex Transcriptional Response. mSystems 3. 10.1128/mSystems.00156-18 PMC6172773 30320218

[35] Stohl EA, Seifert HS (2006) *Neisseria gonorrhoeae* DNA recombination and repair enzymes protect against oxidative damage caused by hydrogen peroxide. J Bacteriol 188: 7645–7651. 10.1128/JB.00801-06 16936020 PMC1636252

[36] Stohl EA, Chan YA, Hackett KT, Kohler PL, Dillard JP, et al (2012) *Neisseria gonorrhoeae* virulence factor NG1686 is a bifunctional M23B family metallopeptidase that influences resistance to hydrogen peroxide and colony morphology. J Biol Chem 287: 11222–11233. 10.1074/jbc.M111.338830 22334697 PMC3322868

[37] Zheng HY, Hassett DJ, Bean K, Cohen MS (1992) Regulation of catalase in *Neisseria gonorrhoeae*. Effects of oxidant stress and exposure to human neutrophils. J Clin Invest 90: 1000–1006. 10.1172/JCI115912 1522209 PMC329956

[38] Turner S, Reid E, Smith H, Cole J (2003) A novel cytochrome c peroxidase from *Neisseria gonorrhoeae*: a lipoprotein from a Gram-negative bacterium. Biochem J 373: 865–873. 10.1042/BJ20030088 12720546 PMC1223530

[39] Tseng HJ, Srikhanta Y, McEwan AG, Jennings MP (2001) Accumulation of manganese in *Neisseria gonorrhoeae* correlates with resistance to oxidative killing by superoxide anion and is independent of superoxide dismutase activity. Mol Microbiol 40: 1175–1186. 10.1046/j.1365-2958.2001.02460.x 11401721

[40] Skaar EP, Tobiason DM, Quick J, Judd RC, Weissbach H, et al (2002) The outer membrane localization of the *Neisseria gonorrhoeae* MsrA/B is involved in survival against reactive oxygen species. Proc Natl Acad Sci U S A 99: 10108–10113. 10.1073/pnas.152334799 12096194 PMC126632

[41] Hofer B, Muller D, Koster H (1985) The pathway of *E*. *coli* RNA polymerase-promoter complex formation as visualized by footprinting. Nucleic Acids Res 13: 5995–6013. 10.1093/nar/13.16.5995 3898021 PMC321928

[42] Carpousis AJ, Gralla JD (1985) Interaction of RNA polymerase with *lacUV5* promoter DNA during mRNA initiation and elongation. Footprinting, methylation, and rifampicin-sensitivity changes accompanying transcription initiation. J Mol Biol 183: 165–177. 10.1016/0022-2836(85)90210-4 2409292

[43] Antunes A, Golfieri G, Ferlicca F, Giuliani MM, Scarlato V, et al (2015) HexR Controls Glucose-Responsive Genes and Central Carbon Metabolism in *Neisseria meningitidis*. J Bacteriol 198: 644–654. 10.1128/JB.00659-15 26644430 PMC4751820

[44] Leyn SA, Li X, Zheng Q, Novichkov PS, Reed S, et al (2011) Control of proteobacterial central carbon metabolism by the HexR transcriptional regulator: a case study in *Shewanella oneidensis*. J Biol Chem 286: 35782–35794. 10.1074/jbc.M111.267963 21849503 PMC3195618

[45] Novichkov PS, Kazakov AE, Ravcheev DA, Leyn SA, Kovaleva GY, et al (2013) RegPrecise 3.0—a resource for genome-scale exploration of transcriptional regulation in bacteria. BMC Genomics 14: 745 10.1186/1471-2164-14-745 24175918 PMC3840689

[46] Reizer J, Hoischen C, Titgemeyer F, Rivolta C, Rabus R, et al (1998) A novel protein kinase that controls carbon catabolite repression in bacteria. Mol Microbiol 27: 1157–1169. 10.1046/j.1365-2958.1998.00747.x 9570401

[47] Gao L, Parsons NJ, Curry A, Cole JA, Smith H (1998) Lactate causes changes in gonococci including increased lipopolysaccharide synthesis during short-term incubation in media containing glucose. FEMS Microbiol Lett 169: 309–316. 10.1111/j.1574-6968.1998.tb13334.x 9868775

[48] Britigan BE, Cohen MS (1986) Effects of human serum on bacterial competition with neutrophils for molecular oxygen. Infect Immun 52: 657–663. 3086230 10.1128/iai.52.3.657-663.1986 PMC260907

[49] Podinovskaia M, Lee W, Caldwell S, Russell DG (2013) Infection of macrophages with *Mycobacterium tuberculosis* induces global modifications to phagosomal function. Cell Microbiol 15: 843–859. 10.1111/cmi.12092 23253353 PMC3620910

[50] van Zwieten R, Wever R, Hamers MN, Weening RS, Roos D (1981) Extracellular proton release by stimulated neutrophils. J Clin Invest 68: 310–313. 10.1172/JCI110250 6265500 PMC370800

[51] Hurst JK, Barrette WC Jr. (1989) Leukocytic oxygen activation and microbicidal oxidative toxins. Crit Rev Biochem Mol Biol 24: 271–328. 10.3109/10409238909082555 2548810

[52] McRipley RJ, Sbarra AJ (1967) Role of the phagocyte in host-parasite interactions. XI. Relationship between stimulated oxidative metabolism and hydrogen peroxide formation, and intracellular killing. J Bacteriol 94: 1417–1424. 4383408 10.1128/jb.94.5.1417-1424.1967 PMC276841

[53] Isabella VM, Clark VL (2011) Deep sequencing-based analysis of the anaerobic stimulon in *Neisseria gonorrhoeae*. BMC Genomics 12: 51 10.1186/1471-2164-12-51 21251255 PMC3032703

[54] Falsetta ML, Steichen CT, McEwan AG, Cho C, Ketterer M, et al (2011) The Composition and Metabolic Phenotype of *Neisseria gonorrhoeae* Biofilms. Front Microbiol 2: 75 10.3389/fmicb.2011.00075 21833322 PMC3153042

[55] Phillips NJ, Steichen CT, Schilling B, Post DM, Niles RK, et al (2012) Proteomic analysis of *Neisseria gonorrhoeae* biofilms shows shift to anaerobic respiration and changes in nutrient transport and outermembrane proteins. PLoS One 7: e38303 10.1371/journal.pone.0038303 22701624 PMC3368942

[56] Falsetta ML, McEwan AG, Jennings MP, Apicella MA (2010) Anaerobic metabolism occurs in the substratum of gonococcal biofilms and may be sustained in part by nitric oxide. Infect Immun 78: 2320–2328. 10.1128/IAI.01312-09 20231417 PMC2863506

[57] Kellogg DS Jr., Peacock WL Jr., Deacon WE, Brown L, Pirkle DI (1963) *Neisseria gonorrhoeae*. I. Virulence Genetically Linked to Clonal Variation. J Bacteriol 85: 1274–1279. 14047217 10.1128/jb.85.6.1274-1279.1963 PMC278328

[58] Dillard JP (2011) Genetic Manipulation of *Neisseria gonorrhoeae*. Curr Protoc Microbiol Chapter 4: Unit4A 2. 10.1002/9780471729259.mc04a02s23 PMC4549065 22045584

[59] Silver LE, Clark VL (1995) Construction of a translational *lacZ* fusion system to study gene regulation in *Neisseria gonorrhoeae*. Gene 166: 101–104. 10.1016/0378-1119(95)00605-6 8529870

[60] Rio DC (2015) Denaturation and electrophoresis of RNA with formaldehyde. Cold Spring Harb Protoc 2015: 219–222. 10.1101/pdb.prot080994 25646498

[61] Edgar R, Domrachev M, Lash AE (2002) Gene Expression Omnibus: NCBI gene expression and hybridization array data repository. Nucleic Acids Res 30: 207–210. 10.1093/nar/30.1.207 11752295 PMC99122

[62] Lee EH, Rouquette-Loughlin C, Folster JP, Shafer WM (2003) FarR regulates the farAB-encoded efflux pump of *Neisseria gonorrhoeae* via an MtrR regulatory mechanism. J Bacteriol 185: 7145–7152. 10.1128/JB.185.24.7145-7152.2003 14645274 PMC296254

[63] Wang H, Ayala JC, Benitez JA, Silva AJ (2012) Interaction of the histone-like nucleoid structuring protein and the general stress response regulator RpoS at *Vibrio cholerae* promoters that regulate motility and hemagglutinin/protease expression. J Bacteriol 194: 1205–1215. 10.1128/JB.05900-11 22194453 PMC3294804

[64] Ayala JC, Wang H, Benitez JA, Silva AJ (2018) Molecular basis for the differential expression of the global regulator VieA in *Vibrio cholerae* biotypes directed by H-NS, LeuO and quorum sensing. Mol Microbiol 107: 330–343. 10.1111/mmi.13884 29152799 PMC5777889

[65] Wang H, Ayala JC, Benitez JA, Silva AJ (2014) The LuxR-type regulator VpsT negatively controls the transcription of *rpoS*, encoding the general stress response regulator, in *Vibrio cholerae* biofilms. J Bacteriol 196: 1020–1030. 10.1128/JB.00993-13 24363348 PMC3957697

[66] Miller JH (1972) Experiments in molecular genetics. Cold Spring Harbor, N.Y: Cold Spring Harbor Laboratory.

[67] Grant JR, Stothard P (2008) The CGView Server: a comparative genomics tool for circular genomes. Nucleic Acids Res 36: W181–184. 10.1093/nar/gkn179 18411202 PMC2447734

[68] Thorvaldsdottir H, Robinson JT, Mesirov JP (2013) Integrative Genomics Viewer (IGV): high-performance genomics data visualization and exploration. Brief Bioinform 14: 178–192. 10.1093/bib/bbs017 22517427 PMC3603213

[69] Hill AV (1910) The possible effects of the aggregation of the molecules of hæmoglobin on its dissociation curves. Journal of Physiology 40 Suppl: iv–vii.

