# Supplementary figures and images for: Transcriptional regulation of a gonococcal gene encoding a virulence factor (L-lactate permease)
Source: PLoS Pathog. 2019 Dec 20;15(12):e1008233. doi: 10.1371/journal.ppat.1008233 (PMC6957213; doi:10.1371/journal.ppat.1008233)

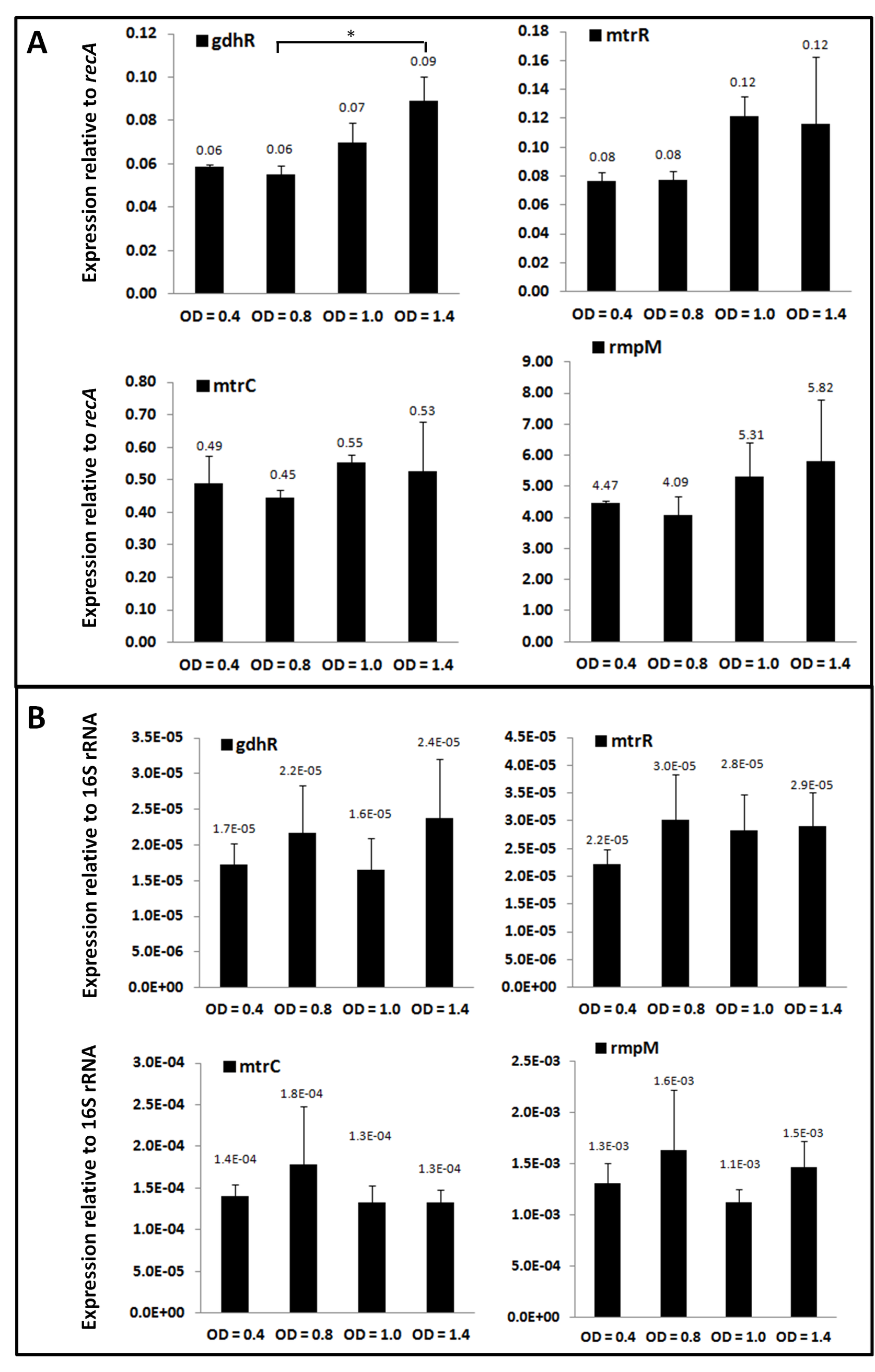

Supplement: S1 Fig — Relative levels of gdhR, mtrR, mtrC and rmpM mRNA were determined by qRT-PCR using recA (A) and 16S rRNA (B) as internal reference genes. Total RNA samples were collected from WT strain FA19 at different optical density (OD) points of its growth in GC broth. Data are presented as the mean (bar) plus the standard deviation (error bar) of 3 biological samples. * represents significant statistical differences at p<0.05 as determined by a non-parametric Kruskal-Wallis test and Dunn posttest. (TIF) [file ppat.1008233.s001.tif]

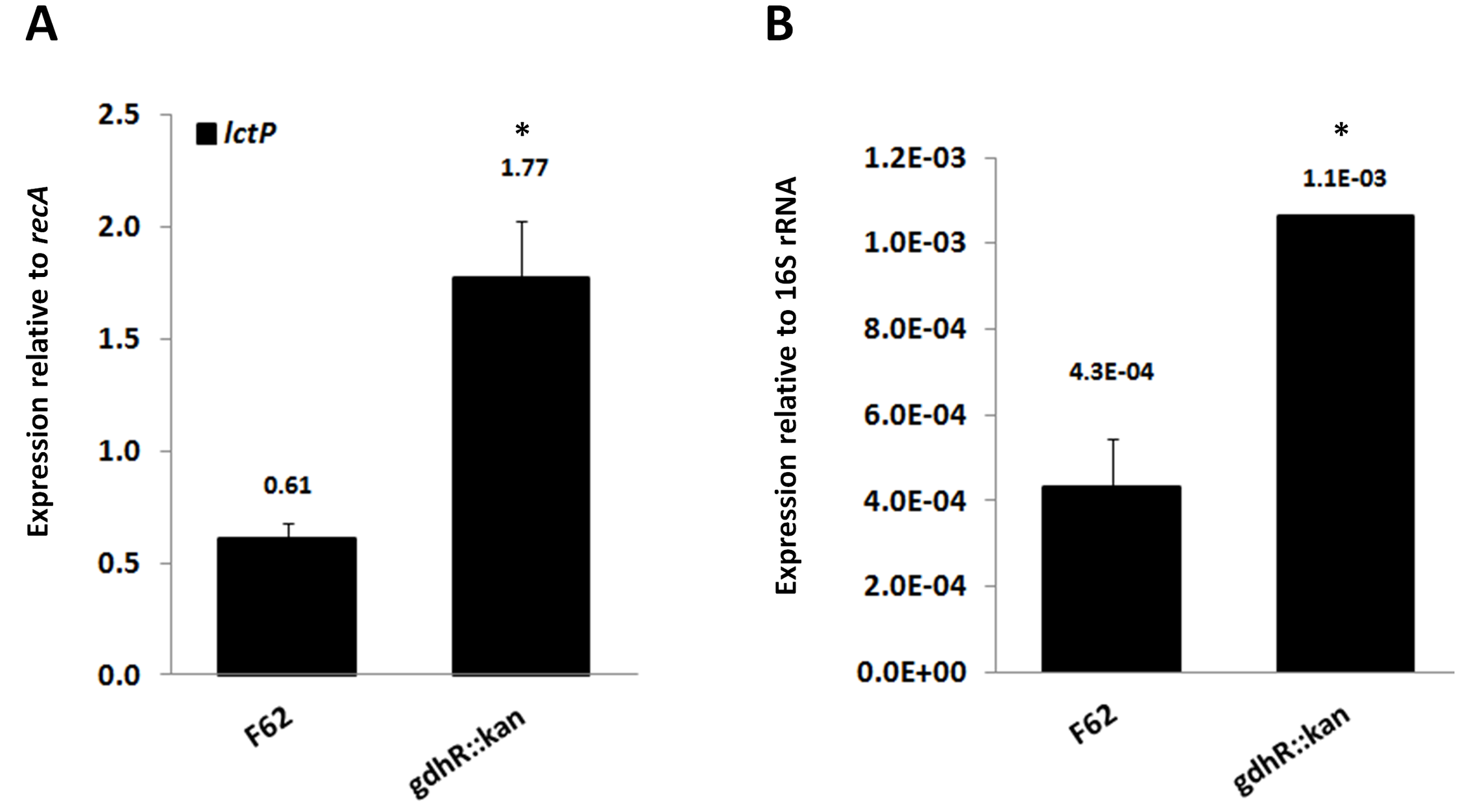

Supplement: S2 Fig — Relative levels of lctP mRNA were determined by qRT-PCR using recA (A) and 16S rRNA (B) as internal reference genes. Total RNA samples were collected from WT strain F62 and its isogenic mutant JC16 (gdhR::kan) grown in GC broth to late-logarithmic phase. Data are presented as the mean (bar) plus the standard deviation (error bar) of 3 biological samples. Significant statistical differences (p<0.01) were determined by a T-test. (TIF) [file ppat.1008233.s002.tif]

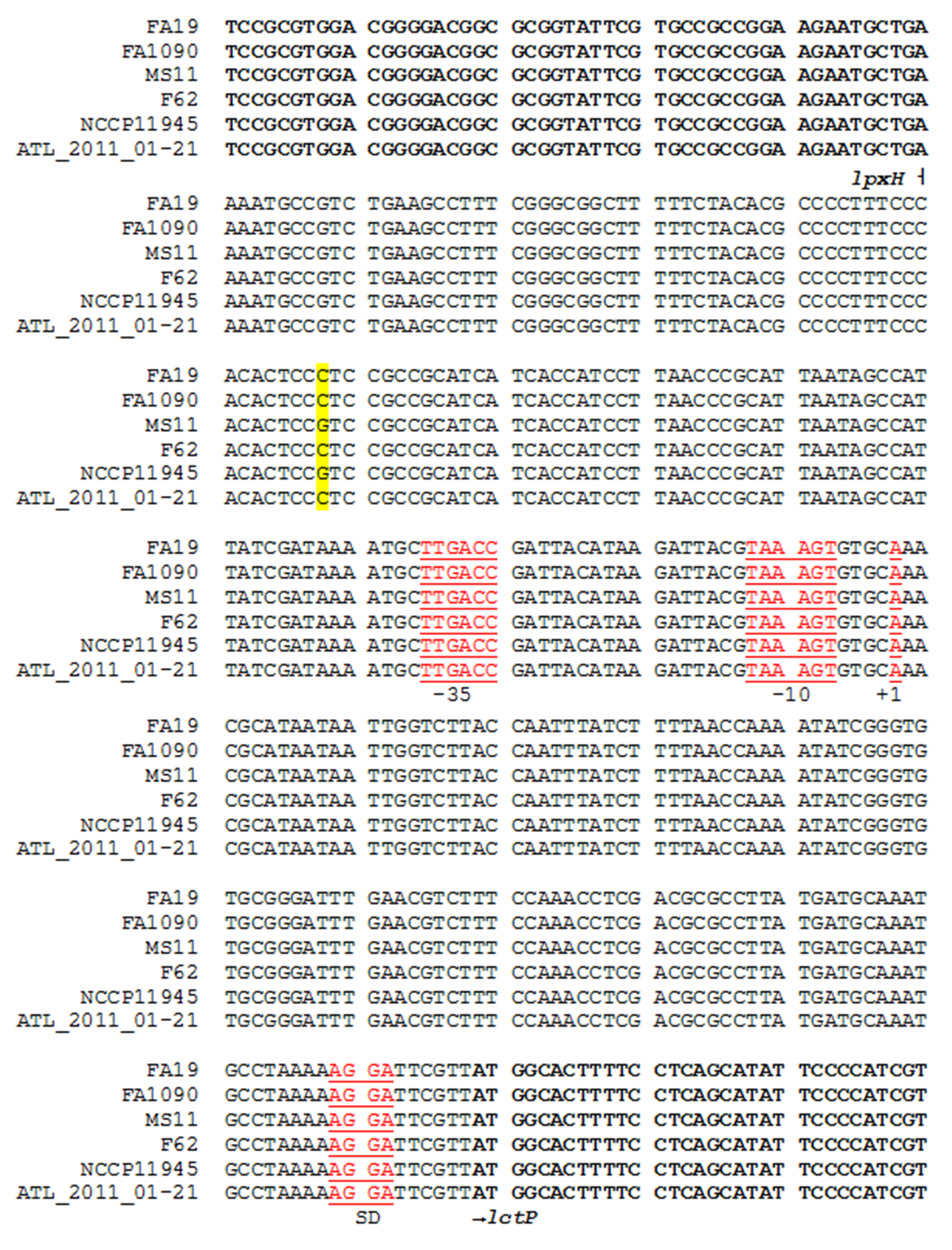

Supplement: S3 Fig — The promoter elements (-35 and -10), the TSS (+1) and Shine-Delgarno (SD) regions are underlined. A G-C polymorphism is highlighted in yellow. The end and start of ORFs corresponding to lpxH and lctP are indicated under the sequence. (TIF) [file ppat.1008233.s003.tif]

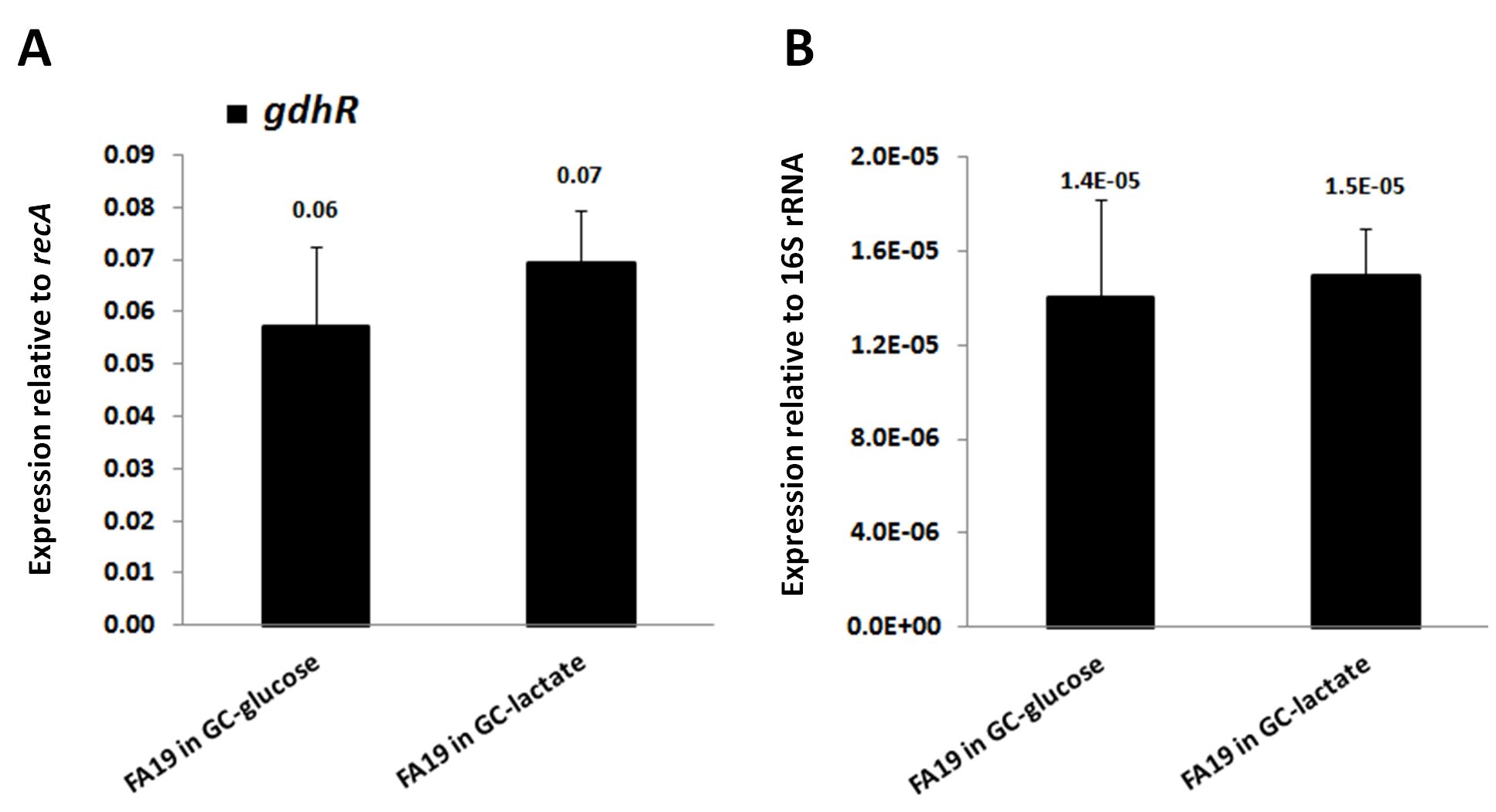

Supplement: S4 Fig — Relative levels of gdhR mRNA were determined by qRT-PCR using recA (A) and 16S rRNA (B) as internal reference genes. Total RNA samples were collected from WT strain FA19 grown to late-logarithmic phase in GC broth supplemented either with D-glucose (22 mM) or L-lactate (22 mM). Data are presented as the mean (bar) plus the standard deviation (error bar) of 4 biological samples. (TIF) [file ppat.1008233.s004.tif]

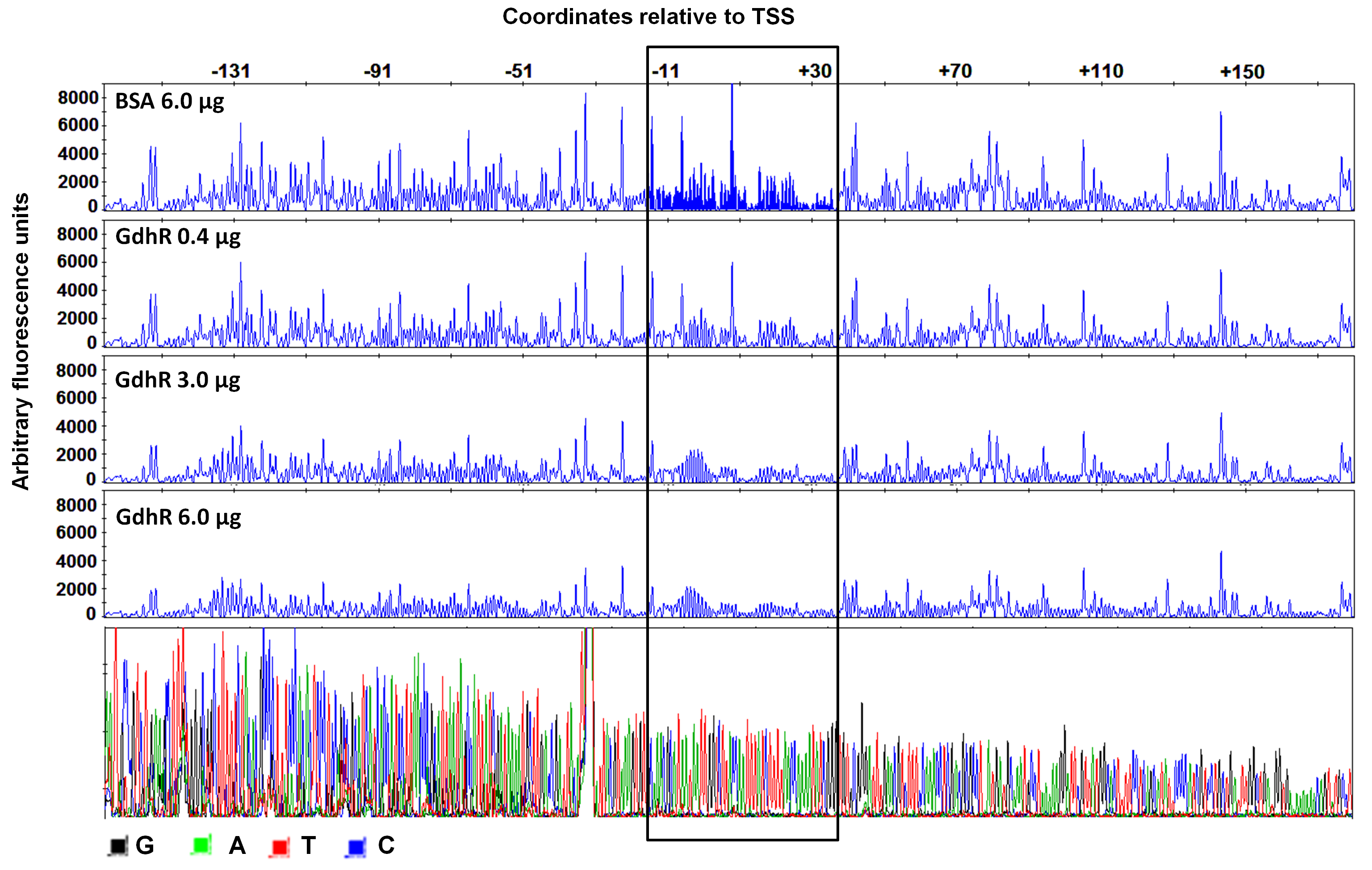

Supplement: S5 Fig — A DNA fragment spanning the lctP promoter region from nucleotide -192 to +381 (relative to the TSS) was fluorescently-labeled with 6-FAM (coding strand) and HEX (template strand) and incubated with BSA (control reaction) or GdhR prior to digestion with DNase I. The DNase I digestion products were analyzed by capillary electrophoresis. The fluorescence signal corresponding to the 6-FAM probe is shown on the y axis of each electropherogram. Fragment coordinates (relative to the TSS) are shown along the top of the BSA electropherogram. Three electropherograms corresponding to 0.4, 3.0 and 6.0 μg of GdhR reactions are shown. The lctP promoter region protected by GdhR is boxed. Dideoxy sequencing reactions were manually-generated using the primer FAM-lctP-DNase and a PCR fragment encoding lctP promoter from -192 to +381 (bottom panel). (TIF) [file ppat.1008233.s005.tif]

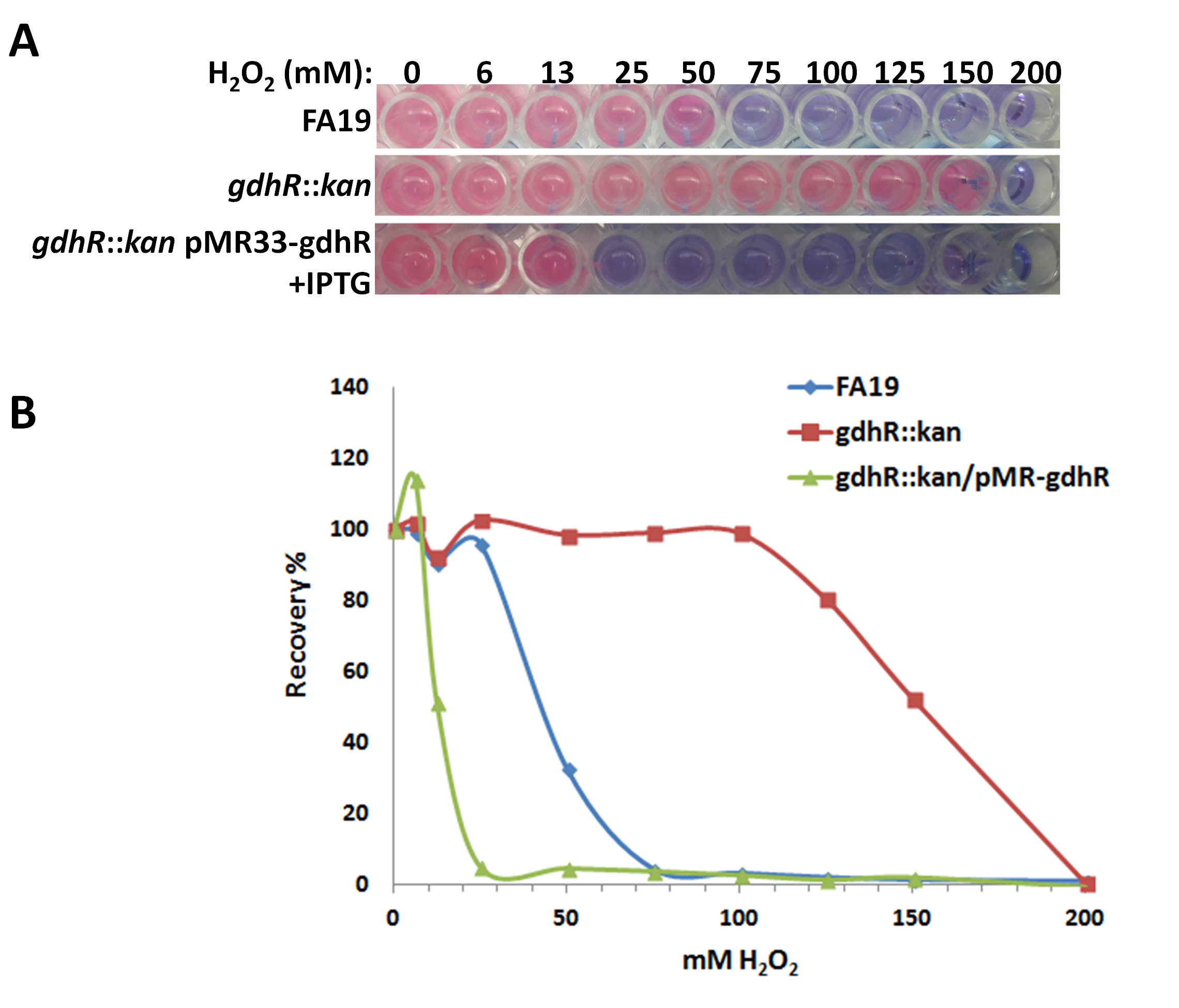

Supplement: S6 Fig — (A) Gonococcal cells (5·108 CFU/mL) of the WT FA19 strain, its isogenic mutant gdhR::kan and gdhR-complemented mutant JC02 (pMR33-gdhR + 1 mM IPTG) were exposed to different concentration of hydrogen peroxide in GC broth and grown overnight in 96-wells plates. Cell viability was determined with the Alamar blue dye. (B) Graphical representation of killing by H2O2 from the fluorescence reading data considering the 0 mM point as 100% growth. Representative experiment of at least two. (TIF) [file ppat.1008233.s006.tif]

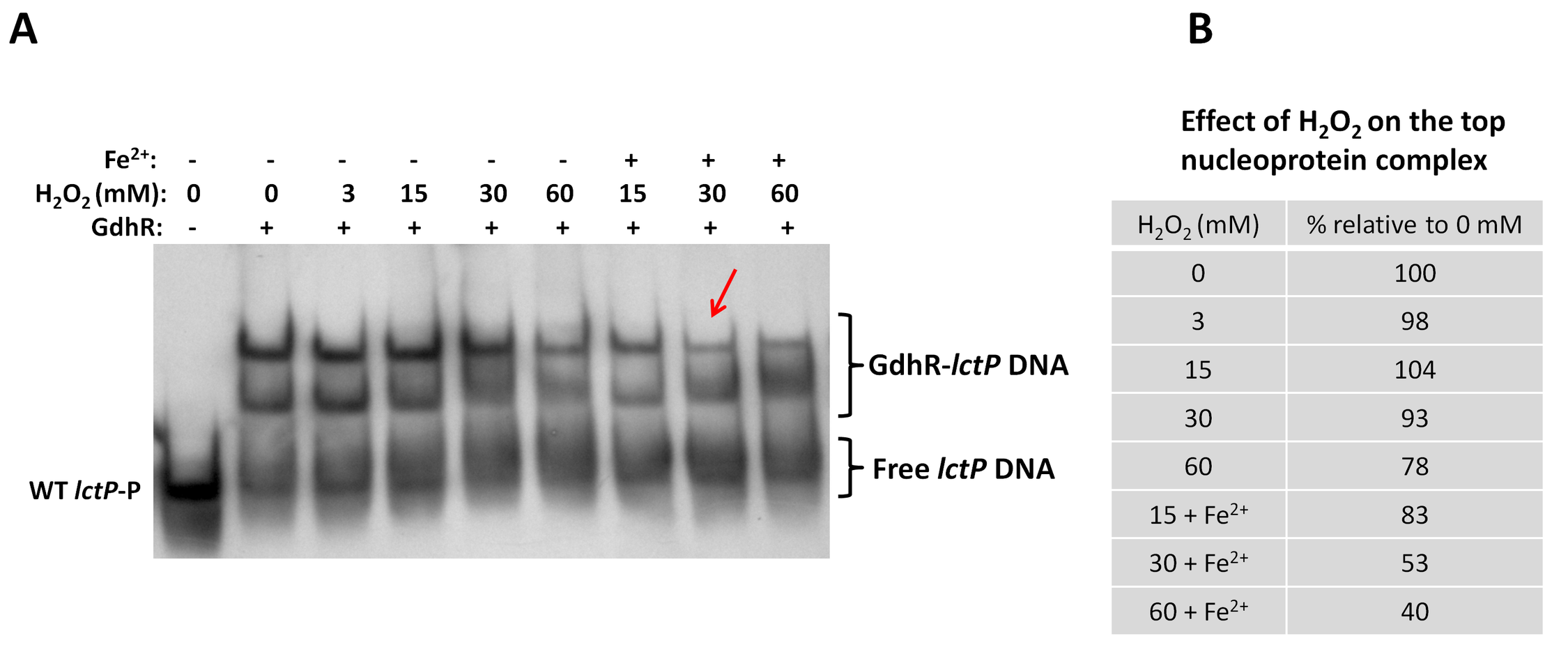

Supplement: S7 Fig — (A) Shown are results from an EMSA experiment that used purified GdhR (120 nanomolar) and a digoxigenin-labeled DNA encompassing the lctP promoter (−313 to −23 relative to the start codon). Binding reactions were performed in the presence of increasing concentrations of either H2O2 alone or H2O2 and 12 μM FeSO4 (Fe2+) that catalyzes the Fenton reaction. The mobility of free DNA and of the nucleoprotein complexes are indicated at the right of the gel. (B) The effect of H2O2 and Fe2+ on the top nucleoprotein complex formation (red arrow) was examined by densitometry of the EMSA gel using the ImageLab 6.0 software. (TIF) [file ppat.1008233.s007.tif]
