# Supplementary material for: Transcriptional regulation of a gonococcal gene encoding a virulence factor (L-lactate permease)
Source: PLoS Pathog. 2019 Dec 20;15(12):e1008233. doi: 10.1371/journal.ppat.1008233 (PMC6957213; doi:10.1371/journal.ppat.1008233)
Supplement: S3 Table — (DOCX) [file ppat.1008233.s010.docx]

**Table S3. Relative expression of genes required for the hydrogen peroxide oxidative damage response in *N. gonorrhoeae***

| **Gene (NGO tag number)** | **FA19** | **FA19 *gdhR*::kan** | **F62** | **F62**  ***gdhR*::kan** | **Reference** |
| --- | --- | --- | --- | --- | --- |
| *recA* (NGO0741) | 34.52 ± 04.41 | 40.18 ± 5.97 | 68.39**^*1^** ± 17.90 | 51.06 ± 10.30 | [[1](#_ENREF_1)] |
| *mpg* (NGO1686) | 1.78 ± 0.73 | 2.11 ± 0.16 | 4.89 ± 1.63 | 4.30 ± 2.20 | [[2](#_ENREF_2)] |
| (NGO0554) | 0.03 ± 0.01 | 0.03 ± 0.01 | 0.40**^*1^** ± 0.12 | 0.65**^*2^** ± 0.13 | [[3](#_ENREF_3)] |
| *katA* (NGO1767) | 34.13 ± 14.57 | 37.34 ± 9.40 | 55.90 ± 20.06 | 35.37 ± 8.59 | [[4](#_ENREF_4)] |
| *ccp* (NGO1769) | 1.17 ± 0.43 | 1.41 ± 0.19 | 0.40**^*1^** ± 0.14 | 0.19 ± 0.10 | [[5](#_ENREF_5)] |
| *mntC* (NGO0168) | 3.64 ± 1.59 | 4.66 ± 0.78 | 5.21 ± 0.74 | 7.82 ± 3.45 | [[6](#_ENREF_6)] |
| *msrA* (NGO2059) | 0.83 ± 0.33 | 0.94 ± 0.22 | 4.87 ± 1.62 | 7.62 ± 4.03 | [[7](#_ENREF_7)] |
| *lctP* (NGO1449) | 9.78 ± 2.67 | 74.54**^*2^** ± 15.80 | 43.14 ± 19.70 | 106.25**^*2^** ± 0.43 | This paper |

**Gonococci were grown in GC broth to late-logarithmic. Relative expression was determined by qRT-PCR using 16S rRNA as internal reference. Values are shown multiplied by 10^5^ relative to 16S rRNA expression levels for easiness and are represented as the mean ± standard deviation of 3 biological samples analyzed in triplicate. Significant statistical differences between the columns were determined using an ANOVA test and Tukey’s post test (at *p* < 0.05).**

**^*1^ statistical differences between the FA19 and F62 WT backgrounds.**

***^2^ statistical differences between the *gdhR* mutants and their respective WT.**

**References**

1. Stohl EA, Seifert HS (2006) Neisseria gonorrhoeae DNA recombination and repair enzymes protect against oxidative damage caused by hydrogen peroxide. J Bacteriol 188: 7645-7651.

2. Stohl EA, Chan YA, Hackett KT, Kohler PL, Dillard JP, et al. (2012) Neisseria gonorrhoeae virulence factor NG1686 is a bifunctional M23B family metallopeptidase that influences resistance to hydrogen peroxide and colony morphology. J Biol Chem 287: 11222-11233.

3. Stohl EA, Criss AK, Seifert HS (2005) The transcriptome response of Neisseria gonorrhoeae to hydrogen peroxide reveals genes with previously uncharacterized roles in oxidative damage protection. Mol Microbiol 58: 520-532.

4. Zheng HY, Hassett DJ, Bean K, Cohen MS (1992) Regulation of catalase in Neisseria gonorrhoeae. Effects of oxidant stress and exposure to human neutrophils. J Clin Invest 90: 1000-1006.

5. Turner S, Reid E, Smith H, Cole J (2003) A novel cytochrome c peroxidase from Neisseria gonorrhoeae: a lipoprotein from a Gram-negative bacterium. Biochem J 373: 865-873.

6. Tseng HJ, Srikhanta Y, McEwan AG, Jennings MP (2001) Accumulation of manganese in Neisseria gonorrhoeae correlates with resistance to oxidative killing by superoxide anion and is independent of superoxide dismutase activity. Mol Microbiol 40: 1175-1186.

7. Skaar EP, Tobiason DM, Quick J, Judd RC, Weissbach H, et al. (2002) The outer membrane localization of the Neisseria gonorrhoeae MsrA/B is involved in survival against reactive oxygen species. Proc Natl Acad Sci U S A 99: 10108-10113.
