# Supplementary material for: Transcriptional regulation of a gonococcal gene encoding a virulence factor (L-lactate permease)
Source: PLoS Pathog. 2019 Dec 20;15(12):e1008233. doi: 10.1371/journal.ppat.1008233 (PMC6957213; doi:10.1371/journal.ppat.1008233)
Supplement: S4 Table — (DOCX) [file ppat.1008233.s011.docx]

**Table S4. Strains and plasmids**

| **Strains** | **Description** | **Reference** |
| --- | --- | --- |
| FA19 | FA19 with point mutation in *rpsL*. Streptomycin resistant | [[1](#_ENREF_1)] |
| F62 | Wild type (kindly provided by S. Morse (Centers for Disease Control and Prevention, Atlanta, GA, USA) | [[2](#_ENREF_2)] |
| FA19 *gdhR::kan* | FA19 with *aphA1* inserted into *gdhR* | [[3](#_ENREF_3)] |
| JC01 | FA19 *gdhR::kan* carrying vector pGCC4-*gdhR* | This study |
| JC02 | FA19 *gdhR::kan* carrying vector pMR33-*gdhR* | This study |
| GP900 | F62 Str^R^ *lctP*::*cat* | [[4](#_ENREF_4)] |
| GP922 | F62 Str^R^ *lctP*::*cat* carrying vector pGCC4-*lctP* | [[4](#_ENREF_4)] |
| JC16 | F62 *gdhR::kan* | This study |
| JC03 | FA19 *lctP*::*cat* | This study |
| JC04 | FA19 *gdhR::kan* *lctP*::*cat* | This study |
| JC05 | F62 *lctP*::*cat* | This study |
| JC24 | F62 *gdhR::kan* *lctP*::*cat* | This study |
| JC28 | FA19 pLES94-*lctP* | This study |
| JC29 | FA19 *gdhR::kan* pLES94-*lctP* | This study |
| JC41 | FA19 *ptsK*::*kan* pLES94-*lctP* | This study |
| **Plasmids** | **Description** | **Reference** |
| pGCC4 | Vector for genetic complementation in gonococci. Carries the *lac* promoter and the *lctP-aspC* loci for homologous recombination. Erm^R^, Km^R^ | [[5](#_ENREF_5)] |
| pMR33 | Vector for genetic complementation in gonococci. Carries the *lac* promoter and the *trpB-iga* loci for homologous recombination. Erm^R^, Km^R^ | [[6](#_ENREF_6)] |
| pGCC4-*gdhR* | pGCC4 with *gdhR* ORF under the *lac* promoter | This study |
| pMR33-*gdhR* | pMR33 with *gdhR* ORF under the *lac* promoter | This study |
| pUC4K | pUC7 carrying a 1.4 Kb HaeII fragment from Tn903 into the PstI site. Km^R^ | [[7](#_ENREF_7)] |
| pLES94 | Vector carrying a promoterless *lacZ* and *proAB* homology regions for recombination in *N. gonorrhoeae* | [[8](#_ENREF_8)] |
| pLES94-*lctP* | *lctP-lacZ* translational fusion in pLES94 | This study |
| pUC18us-*gdhR::kan* | pUC18 carrying a *gdhR* allele inactivated with the Km^R^ cassette from pLG338 | [[3](#_ENREF_3)] |
| pTXB1 | Expression vector for construction of in-frame fusions with the intein/chitin binding domain | New England Biolabs |
| pTXB1-*gdhR* | pTXB1 containing a *gdhR-intein* fusion | This study |
| pUC19-*ptsK* | pUC19 containing a 1156 bp *ptsK* encoding fragment at the EcoRI-SphI sites | This study |
| pUC19-*ptsK*::*kan* | pUC19-*ptsK* containing the Km resistance cassette from pUC4K at the HincII site | This study |

**References**

1. Jerse, A.E., et al., *A gonococcal efflux pump system enhances bacterial survival in a female mouse model of genital tract infection.* Infect Immun, 2003. **71**(10): p. 5576-82.

2. Kellogg, D.S., Jr., et al., *Neisseria Gonorrhoeae. I. Virulence Genetically Linked to Clonal Variation.* J Bacteriol, 1963. **85**: p. 1274-9.

3. Rouquette-Loughlin, C.E., et al., *Control of gdhR Expression in Neisseria gonorrhoeae via Autoregulation and a Master Repressor (MtrR) of a Drug Efflux Pump Operon.* MBio, 2017. **8**(2).

4. Exley, R.M., et al., *Lactate acquisition promotes successful colonization of the murine genital tract by Neisseria gonorrhoeae.* Infect Immun, 2007. **75**(3): p. 1318-24.

5. Mehr, I.J. and H.S. Seifert, *Differential roles of homologous recombination pathways in Neisseria gonorrhoeae pilin antigenic variation, DNA transformation and DNA repair.* Mol Microbiol, 1998. **30**(4): p. 697-710.

6. Ramsey, M.E., et al., *New complementation constructs for inducible and constitutive gene expression in Neisseria gonorrhoeae and Neisseria meningitidis.* Appl Environ Microbiol, 2012. **78**(9): p. 3068-78.

7. Vieira, J. and J. Messing, *The pUC plasmids, an M13mp7-derived system for insertion mutagenesis and sequencing with synthetic universal primers.* Gene, 1982. **19**(3): p. 259-68.

8. Silver, L.E. and V.L. Clark, *Construction of a translational lacZ fusion system to study gene regulation in Neisseria gonorrhoeae.* Gene, 1995. **166**(1): p. 101-4.
