# Supplementary material for: Transcriptional regulation of a gonococcal gene encoding a virulence factor (L-lactate permease)
Source: PLoS Pathog. 2019 Dec 20;15(12):e1008233. doi: 10.1371/journal.ppat.1008233 (PMC6957213; doi:10.1371/journal.ppat.1008233)
Supplement: S5 Table — (DOCX) [file ppat.1008233.s012.docx]

**Table S5. Oligonucleotide primers used in this study**

| **Primer** | **Sequence (5’ → 3’)** |
| --- | --- |
| pac1gepR3 | GATCTTAATTAAGAAGGGTTTTGCAGG |
| pme1gepR4 | GATCGTTTAAACCCAGACCGTCTGAAC |
| pMR33Fw | GCTTCCGGCTCGTATGTTGT |
| NGO1450-F | GGAAGCTTCGAAACGCCGCCATCCATAGC |
| igaRv | TTTGTGCAAGCCAACCTTTATG |
| F1-lctP | ACGCGTCGACAAAGGATTCGTTATGGCACT |
| R1-lctP | ACGCGTCGACTACTACGCCTGAATGCAAAC |
| lctPcheck | AGGCTGGGGCTTGAGCAAAGAC |
| lctP-R2 | CTAATCCCAGATGCCGCATCATT |
| lctPlacZ-F | GAGGATCCCGCGGAAAGGCTGATACACGG |
| lctPlacZ-R | CGGGATCCATAACGAATCCTTTTTAGGCATTT |
| proABFw | AACTCGATGGAAGTGCTGCTGGT |
| lacZRv | AACTGTTGGGAAGGGCGATCGGT |
| gdhR-pTXF | GGAATTCCATATGAAACTGGTAAGG |
| gdhR-pTXR | GTTCGGCTCTTCAGCATACCTCCCAATCCTGC |
| recAqFw | AACCTCGAAGTCATTTCCACCGG |
| recAqRv | TCTGGCATTGGGCGACGGCTTC |
| lctPqFw | CGCCATCAAACTTTTCTACTTCGG |
| lctPqRv | ACATCGATGCAGCCCGTGGTTTC |
| gepR_qRT_F | GCAAAGTTGGCAGGAGCTTT |
| gdhR_qRT_R2 | AACCGAATCCGCTTCAAATCGG |
| GdhR-EMSA-F | GTGGACGGGGACGGCGCG |
| GdhR-EMSA-R | GCATCATAAGGCGCGTCGAG |
| recAP-F | AGCGGCACGGCGATTTTGGT |
| recAP-R | TGCGCCTTTGCCGAAACTTTTT |
| 16Smai-RTF | CCATCGGTATTCCTCCACATCTCT |
| 16Smai-RTR | CGTAGGGTGCGAGCGTTAATC |
| rmpM_qRT_F | AAGCCAAGGTCGCGTAGAAT |
| rmpM_qRT_R | GGCGCGCAATGAATCCTTAT |
| mtrC_qRT_F/ | CGGATTTGGCGCGTTACAAA |
| mtrC_qRT_R | TAATGCGCGAACGGTTCAGA |
| mtrR_qRT_F | CTTGTTTGACGCGTTGTTCCA |
| mtrR_qRT_R | GTGGATGTCGTTGCTTTGCA |
| HEX-lctP-IvT | HEX-TTCACCATCAGCCAAATCAGCAG |
| MotifDel-R | ATTTTGGTTAAAAGATATGCGTTTGCACACTTTAC |
| MotifDel-F | TGTGCAAACGCATATCTTTTAACCAAAATATCGGG |
| FAM-lctP-DNase | 6FAM-GTGGACGGGGACGGCGCG |
| HEX-lctP-DNase | HEX-ACATCGATGCAGCCCGTGGTTTC |
| EcoR-ptsk-F | GAGAATTCGCCGTCTGAAGCCCAGTATCTCCGTCCG |
| Sph-ptsK-R | ATGCATGCGCGCTTGGGCAATGTCTATG |
| pacptsK-F | TATCTTAATTAAATGCCCAGTATCTCCGTCCG |
| pmeptsK-R | ATGTTTAAACGCGCTTGGGCAATGTCTATG |
| 1686qRT-F | AACATCGGAAATACGCGCTGCGC |
| 1686qRT-R | GCGAGTCGCCCGGCTGCACC |
| 0554qRT-F | GATTGCGGGCTGGTTCTCGAAAA |
| 0554qRT-R | CGAAGAAGACATTGGTTGCCGC |
| katAqRT-F | CCATCTGACCATGAACAACGGCG |
| katAqRT-R | GGTAAACGTACCGAACGCGCCC |
| ccp_qRT_F1 | GATGCTTCTCCTGCTTCCGA |
| ccp_qRT_R1 | GTGTCCGAGTTTGACCTGTTCTT |
| mntCqRT-F | GCCACCGCCGCAACTGCCGC |
| mntCqRT-R | AAGTCCCAAGCCGTTGAGCAGGA |
| msrAqRT-F | AAGTTCGGCTGCCTGCTTGCG |
| msrAqRT-R | TTTAATCAGCGTCGGTTTGTCTTTT |

**Underlined sequences correspond to restriction enzyme recognition sites**
