# Supplementary material for: Transcriptional regulation of a gonococcal gene encoding a virulence factor (L-lactate permease)
Source: PLoS Pathog. 2019 Dec 20;15(12):e1008233. doi: 10.1371/journal.ppat.1008233 (PMC6957213; doi:10.1371/journal.ppat.1008233)
Supplement: S1 Appendix — (DOCX) [file ppat.1008233.s013.docx]

**S1 Appendix. Alignment of the *gdhR* allele and upstream sequence from different *N. gonorrhoeae* strains.** Putative Sigma-70 promoter elements and the Shine-Delgarno (SD) sequence are underlined. A transcriptional start site (+1) was estimated using RNA-Seq file corresponding to wild type FA19 strain (GEO sample number GSM3981651) and the IGV software. *gdhR* ORF is in bold font. Single nucleotide polymorphisms are highlighted in yellow.

1 50

FA19 AACCAATTAA AGCAACGTTG CATTACTTTA CGAAACTTTA ATATTCAGGT

FA1090 AACCAATTAA AGCAACGTTG CATTACTTTA CGAAACTTTA ATATTCAGGT

MS11 AACCAATTAA AGCAACGTTG CATTACTTTA CGAAACTTTA ATATTCAGGT

F62 AACCAATTAA AGCAACGTTG CATTACTTTA CGAAACTTTA ATATTCAGGT

51 100

FA19 CAATATATTT TTGGGCGGTT CGGCAGATTT GAATCGGAGC TTTCATTTAA

FA1090 CAATATATTT TTGGGCGGTT CGGCAGATTT GAATCGGAGC TTTCATTTAA

MS11 CAATATATTT TTGGGCGGTT CGGCAGATTT GAATCGGAGC TTTCATTTAA

F62 CAATATATTT TTGGGCGGTT CGGCAGATTT GAATCGGAGC TTTCATTTAA

101 150

FA19 ATCCCGTTAA AACAAATATT TGCATGAACA AAAATTGTAG TTTGGTGTAG

FA1090 ATCCCGTTAA AACAAATATT TGCATGAACA AAAATTGTAG TTTGGTGTAG

MS11 ATCCCGTTAA AACAAATATT TGCATGAACA AAAATTGTAG TTTGGTGTAG

F62 ATCCCGTTAA AACAAATATT TGCATGAACA AAAATTGTAG TTTGGTGTAG

151 200

FA19 TTTTTTCCTG TGTTTCGGGG GCGGTGCGAG GTAAGTGCCG TTGACGGCGG

FA1090 TTTTTTCCTG TGTTTCGGGG GCGGTGCGAG GTAAGTGCCG TTGACGGCGG

MS11 TTTTTTCCTG TGTTTCGGGG GCGGTGCGAG GTAAGTGCCG TTGACGGCGG

F62 TTTTTTCCTG TGTTTCGGGG GCGGTGCGAG GTAAGTGCCG TTGACGGCGG

-35

201 250

FA19 GAACGGCGTG TCATTAGAAT CCGCCCTGAT TGGCCAGTCC AATTTGATGT

FA1090 GAACGGCGTG TCATTAGAAT CCGCCCTGAT TGGCCAGTCC AATTTGATGT

MS11 GAACGGCGTG TCATTAGAAT CCGCCCTGAT TGGCCAGTCC AATTTGATGT

F62 GAACGGCGTG TCATTAGAAT CCGCCCTGAT TGGCCAGTCC AATTTGATGT

-10 +1

251 300

FA19 TTGATGTATT TCGGAAGGGT TCTGCAGGGA ATCTTTATAA AGGAGGAGTG

FA1090 TTGATGTATT TCGGAAGGGT TTTGCAGGGA ATCTTTATAA AGGAGGAGTG

MS11 TTGATGTATT TCGGAAGGGT TCTGCAGGGA ATCTTTATAA AGGAGGAGTG

F62 TTGATGTATT TCGGAAGGGT TCTGCAGGGA ATCTTTATAA AGGAGGAGTG

SD

301 350

FA19 **ATGAAACTGG TAAGGCCTCA GAAAATCAGC GATCAGGTAT TGTCGGTATT**

FA1090 **ATGAAACTGG TAAGGCCTCA GAAAATCAGC GATCAGGTAT TGTCGGTATT**

MS11 **ATGAAACTGG TAAGGTCTCA GAAAATCAGC GATCAGGTAT TGTCGGTATT**

F62  **ATGAAACTGG TAAGGCCTCA GAAAATCAGC GATCAGGTAT TGTCGGTATT**

**→** *gdhR*

351 400

FA19 **GGAAGGCCGC ATCGTGGAAG GGGTTTACGC GGAAGGGGGC AAGATTCCGC**

FA1090 **GGAAGGCCGT ATCGTGGAAG GGGTTTACGC GGAAGGGGGC AAGATTCCGC**

MS11 **GGAAGGCCGC ATCGTGGAAG GGGTTTACGC GGAAGGGGGC AAGATTCCGC**

F62 **GGAAGGCCGT ATCGTGGAAG GGGTTTACGC GGAAGGGGGC AAGATTCCGC**

401 450

FA19 **CCGAACGCGT TTTGGCGGAA GAGTTCGGCG TTTCGCGTCC GTCGGTCCGG**

FA1090 **CCGAACGCGT TTTGGCGGAA GAGTTCGGCG TTTCGCGTCC GTCGGTCCGG**

MS11 **CCGAACGCGT TTTGGCGGAA GAGTTCGGCG TTTCGCGTCC GTCGGTCCGG**

F62 **CCGAACGCGT TTTGGCGGAA GAGTTCGGCG TTTCGCGTCC GTCGGTCCGG**

451 500

FA19 **TCGGCATTGA ATATTTTGGT TGCCCGTCAG ATTTTGGAAG CGAGGCAGGG**

FA1090 **TCGGCATTGA ATATTTTGGT TGCCCGTCAG ATTTTGGAAG CGAGGCAGGG**

MS11 **TCGGCATTGA ATATTTTGGT TGCCCGTCAG ATTTTGGAAG CGAGGCAGGG**

F62 **TCGGCATTGA ATATTTTGGT TGCCCGTCAG ATTTTGGAAG CGAGGCAGGG**

501 550

FA19 **CGATGGTTAT TATGTTTCGG TCAAGCCGCA GCAGGATTTT TTGCAAAGTT**

FA1090 **CGATGGTTAT TATGTTTCGG TCAAGCCGCA GCAGGATTTT TTGCAAAGTT**

MS11 **CGATGGTTAT TATGTTTCGG TCAAGCCGCA GCAGGATTTT TTGCAAAGTT**

F62 **CGATGGTTAT TATGTTTCGG TCAAGCCGCA GCAGGATTTT TTGCAAAGTT**

551 600

FA19 **GGCAGGAGCT TTTGGGCAAA CATTCCAATT GGGAACAGGA TGTTTTTGAT**

FA1090 **GGCAGGAGCT TTTGGGCAAA CATTCCAATT GGGAACAGGA TGTTTTTGAT**

MS11 **GGCAGGAGCT TTTGGGCAAA CATTCCAATT GGGAACAGGA TGTTTTTGAT**

F62 **GGCAGGAGCT TTTGGGCAAA CATTCCAATT GGGAACAGGA TGTTTTTGAT**

601 650

FA19 **TTCAGCTGCC ACATCGAGGG CTGTATGGCG GCATTGGCGG CAGAACGCCG**

FA1090 **TTCAGCTGCC ACATCGAGGG CTGTATGGCG GCATTGGCGG CAGAACGCCG**

MS11 **TTCAGCTGCC ACATCGAGGG CTGTATGGCG GCATTGGCGG CAGAACGCCG**

F62 **TTCAGCTGCC ACATCGAGGG CTGTATGGCG GCATTGGCGG CAGAACGCCG**

651 700

FA19 **GACGGATGCC GATTTGAAGC GGATTCGGTT TTGGCTTGAA AAGTTTGAGG**

FA1090 **GACGGATGCC GATTTGAAGC GGATTCGGTT TTGGCTTGAA AAGTTTGAGG**

MS11 **GACGGATGCC GATTTGAAGC GGATTCGGTT TTGGCTTGAA AAGTTTGAGG**

F62 **GACGGATGCC GATTTGAAGC GGATTCGGTT TTGGCTTGAA AAGTTTGAGG**

701 750

FA19 **AAGCGTGCGG AAGCGGCAAT CTGGAACATC AGGGCGAAGC CGATGTCAGC**

FA1090 **AAGCGTGCGG AAGCGGCAAT CTGGAACATC AGGGCGAAGC CGATGTCAGC**

MS11 **AAGCGTGCGG AAGCGGCAAT CTGGAACATC AGGGCGAAGC CGATGTCAGC**

F62 **AAGCGTGCGG AAGCGGCAAT CTGGAACATC AGGGCGAAGC CGATGTCAGC**

751 800

FA19 **TTCCATCAGA CGATTGCGGA TGCGGCGCAC AATTTGTTGT TCAGCCATTT**

FA1090 **TTCCATCAGA CGATTGCGGA TGCGGCGCAC AATTTGTTGT TCAGCCATTT**

MS11 **TTCCATCAGA CGATTGCGGA TGCGGCGCAC AATTTGTTGT TCAGCCATTT**

F62 **TTCCATCAGA CGATTGCGGA TGCGGCGCAC AATTTGTTGT TCAGCCATTT**

801 850

FA19 **GTCGGGCGGT TTGTTGAAAA TGCTGTACCG GCAGACGCGC AGCAGCCTTA**

FA1090 **GTCGGGCGGT TTGTTGAAAA TGCTGTACCG GCAGACGCGC AGCAGCCTTA**

MS11 **GTCGGGCGGT TTGTTGAAAA TGCTGTACCG GCAGACGCGC AGCAGCCTTA**

F62 **GTCGGGCGGT TTGTTGAAAA TGCTGTACCG GCAGACGCGC AGCAGCCTTA**

851 900

FA19 **TTTATCTGAA TCAGGAAGAA GATCCGCGTC CGAAACTGAT GGCGCAGCAC**

FA1090 **TTTATCTGAA TCAGGAAGAA GATCCGCGTC CGAAACTGAT GGCGCAGCAC**

MS11 **TTTATCTGAA TCAGGAAGAA GATCCGCGTC CGAAACTGAT GGCGCAGCAC**

F62 **TTTATCTGAA TCAGGAAGAA GATCCGCGTC CGAAACTGAT GGCGCAGCAC**

901 950

FA19 **CGCGTGCTGT ATGAGGCGAT ATCGAACCGC CGGCCGGGCG AGGCTTCGGA**

FA1090 **CGCGTGCTGT ATGAGGCGAT ATCGAACCGC CGGCCGGGCG AGGCTTCGGA**

MS11 **CGCGTGCTGT ATGAGGCGAT ATCGAACCGC CGGCCGGGCG AGGCTTCGGA**

F62 **CGCGTGCTGT ATGAGGCGAT ATCGAACCGC CGGCCGGGCG AGGCTTCGGA**

951 1000

FA19 **GGCGGCAAAG GCTCATTTGA ATTATGTGGC GAGCAGCATA TTGAAAGACA**

FA1090 **GGCGGCAAAG GCTCATTTGA ATTATGTGGC GAGCAGCATA TTGAAAGACA**

MS11 **GGCGGCAAAG GCTCATTTGA ATTATGTGGC GAGCAGCATA TTGAAAGACA**

F62 **GGCGGCAAAG GCTCATTTGA ATTATGTGGC GAGCAGCATA TTGAAAGACA**

1001 1050

FA19 **GGGAATATCA GAGCCGTAAC CGCCACGCGG ATACTTTGGC GCAAAACGAT**

FA1090 **GGGAATATCA GAGCCGTAAC CGCCACGCGG ATACTTTGGC GCAAAACGAT**

MS11 **GGGAATATCA GAGCCGTAAC CGCCACGCGG ATACTTTGGC GCAAAACGAT**

F62 **GGGAATATCA GAGCCGTAAC CGCCACGCGG ATACTTTGGC GCAAAACGAT**

1051 1080

FA19 **TTGAAGCGCG TGCAGGATTG GGAGGTATGA**

FA1090 **TTGAAGCGCG TGCAGGATTG GGAGGTATGA**

MS11 **TTGAAGCGCG TGCAGGATTG GGAGGTATGA**

F62 **TTGAAGCGCG TGCAGGATTG GGAGGTATGA**

*gdhR* **˧**
