# Supplementary material for: Transcriptional regulation of a gonococcal gene encoding a virulence factor (L-lactate permease)
Source: PLoS Pathog. 2019 Dec 20;15(12):e1008233. doi: 10.1371/journal.ppat.1008233 (PMC6957213; doi:10.1371/journal.ppat.1008233)

**S2 Appendix. Sensitivity of *N. gonorrhoeae* strains to hydrogen peroxide in GC broth.** Gonococci from wild type strains FA19 and F62 were incubated overnight at 37^o^C with or without H_2_O_2_ before their viability was assessed by spot plating of 10 µL culture on GC agar plates. Cell densities were adjusted to an OD_600_ 0.05 at the beginning of the experiment in GC broth containing D-glucose (22 mM) and L-lactate (3 mM).


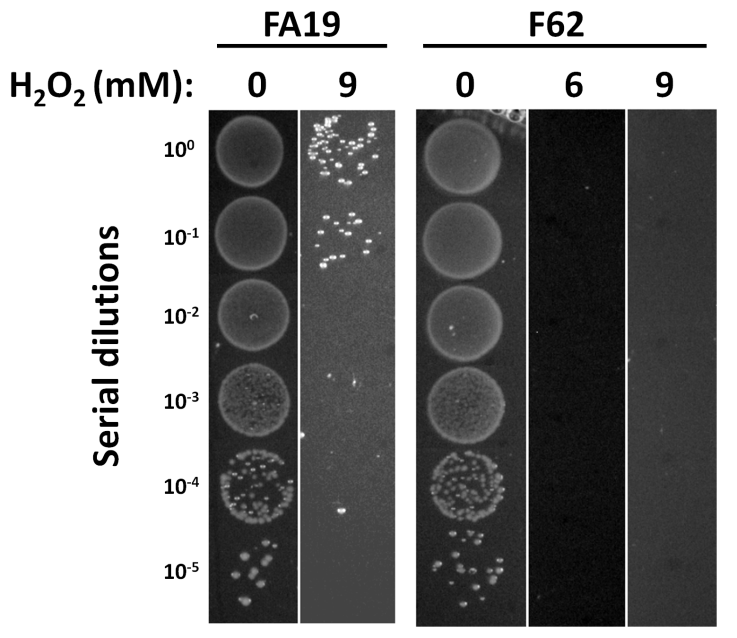

Supplement: S2 Appendix — (DOCX) [file ppat.1008233.s014.docx]
