# Supplementary material for: Transcriptional regulation of a gonococcal gene encoding a virulence factor (L-lactate permease)
Source: PLoS Pathog. 2019 Dec 20;15(12):e1008233. doi: 10.1371/journal.ppat.1008233 (PMC6957213; doi:10.1371/journal.ppat.1008233)
Supplement: S3 Appendix — (DOCX) [file ppat.1008233.s015.docx]

**S3 Appendix. Bioinformatic detection of the *N. meningitidis* HexR DNA-binding motif within the *N. gonorrhoeae* FA1090 genome using the FIMO algorithm (Find Individual Motif Occurrences [1]).**

- ***Neisseria meningitidis* HexR DNA-binding motif used for analysis (reported in [2])**

KTGTANTWWWANTACAM or [GT]TGTA[AG]T[AT][AT][TA]A[TC]TACA[AC]

- **FIMO analysis results**

| **Alt ID** | **Sequence Name** | **Strand** | **Start** | **End** | **p-value** | **q-value** | **Matched Sequence** |
| --- | --- | --- | --- | --- | --- | --- | --- |
| KTGTANTWWWANTACAM | YP_209091.1\|NGO2078 | + | 16 | 32 | 1.83e-07 | 0.0449 | CTGTAATAAAAGTACAA |
| KTGTANTWWWANTACAM | YP_209091.1\|NGO2078 | - | 16 | 32 | 1.83e-07 | 0.0449 | TTGTACTTTTATTACAG |
| KTGTANTWWWANTACAM | YP_209090.1\|NGO2077 | + | 309 | 325 | 1.83e-07 | 0.0449 | TTGTACTTTTATTACAG |
| KTGTANTWWWANTACAM | YP_209090.1\|NGO2077 | - | 309 | 325 | 1.83e-07 | 0.0449 | CTGTAATAAAAGTACAA |
| KTGTANTWWWANTACAM | YP_207846.1\|NGO0714 | + | 102 | 118 | 2.45e-07 | 0.0449 | GAGTAGTAATACTACAC |
| KTGTANTWWWANTACAM | YP_207846.1\|NGO0714 | - | 102 | 118 | 2.45e-07 | 0.0449 | GTGTAGTATTACTACTC |
| KTGTANTWWWANTACAM | YP_207847.1\|NGO0715 | + | 402 | 418 | 2.45e-07 | 0.0449 | GTGTAGTATTACTACTC |
| KTGTANTWWWANTACAM | YP_207847.1\|NGO0715 | - | 402 | 418 | 2.45e-07 | 0.0449 | GAGTAGTAATACTACAC |
| KTGTANTWWWANTACAM | YP_208032.1\|NGO0924 | + | 168 | 184 | 1.07e-06 | 0.0971 | TTGTTTTTAAAATACAC |
| KTGTANTWWWANTACAM | YP_208032.1\|NGO0924 | - | 168 | 184 | 1.07e-06 | 0.0971 | GTGTATTTTAAAAACAA |
| KTGTANTWWWANTACAM | YP_208028.1\|sdhB | + | 66 | 82 | 1.18e-06 | 0.0971 | TTGAATTTATAATACAA |
| KTGTANTWWWANTACAM | YP_208028.1\|sdhB | - | 66 | 82 | 1.18e-06 | 0.0971 | TTGTATTATAAATTCAA |
| KTGTANTWWWANTACAM | YP_207847.1\|NGO0715 | + | 246 | 262 | 1.18e-06 | 0.0971 | TTGTAATTTTGTTACAA |
| KTGTANTWWWANTACAM | YP_207846.1\|NGO0714 | + | 258 | 274 | 1.18e-06 | 0.0971 | TTGTAACAAAATTACAA |
| KTGTANTWWWANTACAM | YP_207847.1\|NGO0715 | - | 246 | 262 | 1.18e-06 | 0.0971 | TTGTAACAAAATTACAA |
| KTGTANTWWWANTACAM | YP_207846.1\|NGO0714 | - | 258 | 274 | 1.18e-06 | 0.0971 | TTGTAATTTTGTTACAA |
| KTGTANTWWWANTACAM | YP_207920.1\|NGO0794 | + | 354 | 370 | 1.19e-06 | 0.0971 | TTGTATTTTAAATAGAA |
| KTGTANTWWWANTACAM | YP_207920.1\|NGO0794 | - | 354 | 370 | 1.19e-06 | 0.0971 | TTCTATTTAAAATACAA |
| KTGTANTWWWANTACAM | YP_208600.1\|NGO1552 | + | 65 | 81 | 6.31e-06 | 0.421 | TTGTACTTTCATTAGAA |
| KTGTANTWWWANTACAM | YP_208599.1\|NGO1551 | + | 81 | 97 | 6.31e-06 | 0.421 | TTCTAATGAAAGTACAA |
| KTGTANTWWWANTACAM | YP_208600.1\|NGO1552 | - | 65 | 81 | 6.31e-06 | 0.421 | TTCTAATGAAAGTACAA |
| KTGTANTWWWANTACAM | YP_208599.1\|NGO1551 | - | 81 | 97 | 6.31e-06 | 0.421 | TTGTACTTTCATTAGAA |
| KTGTANTWWWANTACAM | YP_208849.1\|NGO1820 | + | 187 | 203 | 1.34e-05 | 0.515 | TTCTCGTAAAAGTACAC |
| KTGTANTWWWANTACAM | YP_208849.1\|NGO1820 | - | 187 | 203 | 1.34e-05 | 0.515 | GTGTACTTTTACGAGAA |
| KTGTANTWWWANTACAM | YP_207847.1\|NGO0715 | + | 226 | 242 | 1.34e-05 | 0.515 | GTGCTATTATAGTACAA |
| KTGTANTWWWANTACAM | YP_207847.1\|NGO0715 | - | 226 | 242 | 1.34e-05 | 0.515 | TTGTACTATAATAGCAC |
| KTGTANTWWWANTACAM | YP_208842.1\|NGO1812 | + | 261 | 277 | 1.34e-05 | 0.515 | GTGTTGTAAAAATGCAA |
| KTGTANTWWWANTACAM | YP_208842.1\|NGO1812 | - | 261 | 277 | 1.34e-05 | 0.515 | TTGCATTTTTACAACAC |
| KTGTANTWWWANTACAM | YP_207846.1\|NGO0714 | + | 278 | 294 | 1.34e-05 | 0.515 | TTGTACTATAATAGCAC |
| KTGTANTWWWANTACAM | YP_207846.1\|NGO0714 | - | 278 | 294 | 1.34e-05 | 0.515 | GTGCTATTATAGTACAA |
| KTGTANTWWWANTACAM | YP_208993.1\|NGO1972 | + | 77 | 93 | 1.74e-05 | 0.515 | GTGGATTAAATTTACAA |
| KTGTANTWWWANTACAM | YP_208993.1\|NGO1972 | - | 77 | 93 | 1.74e-05 | 0.515 | TTGTAAATTTAATCCAC |
| KTGTANTWWWANTACAM | YP_208976.1\|NGO1953 | + | 125 | 141 | 1.74e-05 | 0.515 | TTGTCATTTAAATACCC |
| KTGTANTWWWANTACAM | YP_208080.1\|NGO0982 | + | 134 | 150 | 1.74e-05 | 0.515 | GTGTAAATTTTTTACAA |
| KTGTANTWWWANTACAM | YP_208976.1\|NGO1953 | - | 125 | 141 | 1.74e-05 | 0.515 | GGGTATTTAAATGACAA |
| KTGTANTWWWANTACAM | YP_207625.1\|NGO0475 | + | 147 | 163 | 1.74e-05 | 0.515 | TAATAGTTATAATACAC |
| KTGTANTWWWANTACAM | YP_208080.1\|NGO0982 | - | 134 | 150 | 1.74e-05 | 0.515 | TTGTAAAAAATTTACAC |
| KTGTANTWWWANTACAM | YP_207625.1\|NGO0475 | - | 147 | 163 | 1.74e-05 | 0.515 | GTGTATTATAACTATTA |
| KTGTANTWWWANTACAM | YP_207470.1\|NGO0311 | + | 228 | 244 | 1.74e-05 | 0.515 | TTGTATTTTAACTATCC |
| KTGTANTWWWANTACAM | YP_207470.1\|NGO0311 | - | 228 | 244 | 1.74e-05 | 0.515 | GGATAGTTAAAATACAA |
| KTGTANTWWWANTACAM | YP_208164.1\|NGO1085 | + | 21 | 37 | 1.92e-05 | 0.515 | GTTTATTATTCTTACAA |
| KTGTANTWWWANTACAM | YP_208164.1\|NGO1085 | - | 21 | 37 | 1.92e-05 | 0.515 | TTGTAAGAATAATAAAC |
| KTGTANTWWWANTACAM | YP_207489.1\|NGO0331 | + | 205 | 221 | 1.92e-05 | 0.515 | TTGGAAGAAAAATACAA |
| KTGTANTWWWANTACAM | YP_207489.1\|NGO0331 | - | 205 | 221 | 1.92e-05 | 0.515 | TTGTATTTTTCTTCCAA |
| KTGTANTWWWANTACAM | YP_208837.1\|NGO1807 | + | 428 | 444 | 1.92e-05 | 0.515 | GTGTTTTAAAAATAAAA |
| KTGTANTWWWANTACAM | YP_208837.1\|NGO1807 | - | 428 | 444 | 1.92e-05 | 0.515 | TTTTATTTTTAAAACAC |
| KTGTANTWWWANTACAM | YP_207464.1\|ihfA | + | 16 | 32 | 1.96e-05 | 0.515 | TTGAATTTTAAATAAAA |
| KTGTANTWWWANTACAM | YP_207464.1\|ihfA | - | 16 | 32 | 1.96e-05 | 0.515 | TTTTATTTAAAATTCAA |
| KTGTANTWWWANTACAM | YP_208010.1\|NGO0900 | + | 82 | 98 | 1.96e-05 | 0.515 | TTAAAATAAAAATACAA |
| KTGTANTWWWANTACAM | YP_208010.1\|NGO0900 | - | 82 | 98 | 1.96e-05 | 0.515 | TTGTATTTTTATTTTAA |
| KTGTANTWWWANTACAM | YP_208878.1\|rpsL | + | 106 | 122 | 1.96e-05 | 0.515 | TTGCAATAAAAATATAA |
| KTGTANTWWWANTACAM | YP_208878.1\|rpsL | - | 106 | 122 | 1.96e-05 | 0.515 | TTATATTTTTATTGCAA |
| KTGTANTWWWANTACAM | YP_208588.1\|NGO1540 | + | 186 | 202 | 1.96e-05 | 0.515 | TTGTATTTTTATTATTA |
| KTGTANTWWWANTACAM | YP_208588.1\|NGO1540 | - | 186 | 202 | 1.96e-05 | 0.515 | TAATAATAAAAATACAA |
| KTGTANTWWWANTACAM | YP_208009.1\|greA | + | 293 | 309 | 1.96e-05 | 0.515 | TTGTATTTTTATTTTAA |
| KTGTANTWWWANTACAM | YP_208009.1\|greA | - | 293 | 309 | 1.96e-05 | 0.515 | TTAAAATAAAAATACAA |
| KTGTANTWWWANTACAM | YP_208955.1\|NGO1930 | + | 48 | 64 | 2.77e-05 | 0.635 | CTGAAATAAAACTACAT |
| KTGTANTWWWANTACAM | YP_208955.1\|NGO1930 | - | 48 | 64 | 2.77e-05 | 0.635 | ATGTAGTTTTATTTCAG |
| KTGTANTWWWANTACAM | YP_207533.1\|NGO0377 | + | 104 | 120 | 2.77e-05 | 0.635 | ATATATTTACACTACAC |
| KTGTANTWWWANTACAM | YP_207533.1\|NGO0377 | - | 104 | 120 | 2.77e-05 | 0.635 | GTGTAGTGTAAATATAT |
| KTGTANTWWWANTACAM | YP_209024.1\|NGO2004 | + | 169 | 185 | 2.77e-05 | 0.635 | ATGTATTCAAACGACAA |
| KTGTANTWWWANTACAM | YP_209024.1\|NGO2004 | - | 169 | 185 | 2.77e-05 | 0.635 | TTGTCGTTTGAATACAT |
| KTGTANTWWWANTACAM | YP_208956.1\|NGO1931 | + | 370 | 386 | 2.77e-05 | 0.635 | ATGTAGTTTTATTTCAG |
| KTGTANTWWWANTACAM | YP_208956.1\|NGO1931 | - | 370 | 386 | 2.77e-05 | 0.635 | CTGAAATAAAACTACAT |
| KTGTANTWWWANTACAM | YP_208071.1\|NGO0973 | + | 17 | 33 | 5.92e-05 | 0.967 | TTGTCCCCAAAATACAC |
| KTGTANTWWWANTACAM | YP_208071.1\|NGO0973 | - | 17 | 33 | 5.92e-05 | 0.967 | GTGTATTTTGGGGACAA |
| KTGTANTWWWANTACAM | YP_208649.1\|NGO1609 | + | 65 | 81 | 5.92e-05 | 0.967 | GGGTAGTTTTGTTACAG |
| KTGTANTWWWANTACAM | YP_208649.1\|NGO1609 | - | 65 | 81 | 5.92e-05 | 0.967 | CTGTAACAAAACTACCC |
| KTGTANTWWWANTACAM | YP_207238.1\|NGO0062 | + | 301 | 317 | 5.92e-05 | 0.967 | GTGTAGGTCAAAAACAA |
| KTGTANTWWWANTACAM | YP_207238.1\|NGO0062 | - | 301 | 317 | 5.92e-05 | 0.967 | TTGTTTTTGACCTACAC |
| KTGTANTWWWANTACAM | YP_208333.1\|NGO1262 | + | 383 | 399 | 5.92e-05 | 0.967 | ATGAAGTTTTAGTAAAC |
| KTGTANTWWWANTACAM | YP_208242.1\|NGO1164 | + | 383 | 399 | 5.92e-05 | 0.967 | ATGAAGTTTTAGTAAAC |
| KTGTANTWWWANTACAM | YP_208242.1\|NGO1164 | - | 383 | 399 | 5.92e-05 | 0.967 | GTTTACTAAAACTTCAT |
| KTGTANTWWWANTACAM | YP_208333.1\|NGO1262 | - | 383 | 399 | 5.92e-05 | 0.967 | GTTTACTAAAACTTCAT |
| KTGTANTWWWANTACAM | YP_208255.1\|NGO1183 | + | 11 | 27 | 8.06e-05 | 0.967 | TTGAACAAACAATACAA |
| KTGTANTWWWANTACAM | YP_208037.1\|metF | + | 18 | 34 | 8.06e-05 | 0.967 | GTGTTATAACATAACAA |
| KTGTANTWWWANTACAM | NGO_t41\|NGO_t41 | + | 26 | 42 | 8.06e-05 | 0.967 | CGGTATTTTTTATACAC |
| KTGTANTWWWANTACAM | YP_208255.1\|NGO1183 | - | 11 | 27 | 8.06e-05 | 0.967 | TTGTATTGTTTGTTCAA |
| KTGTANTWWWANTACAM | YP_208037.1\|metF | - | 18 | 34 | 8.06e-05 | 0.967 | TTGTTATGTTATAACAC |
| KTGTANTWWWANTACAM | YP_208195.1\|NGO1116 | + | 39 | 55 | 8.06e-05 | 0.967 | ACGTACTTATAATGCAA |
| KTGTANTWWWANTACAM | NGO_t41\|NGO_t41 | - | 26 | 42 | 8.06e-05 | 0.967 | GTGTATAAAAAATACCG |
| KTGTANTWWWANTACAM | YP_208372.1\|NGO1308 | + | 53 | 69 | 8.06e-05 | 0.967 | TTGAAATGAAAAAACAC |
| KTGTANTWWWANTACAM | YP_208195.1\|NGO1116 | - | 39 | 55 | 8.06e-05 | 0.967 | TTGCATTATAAGTACGT |
| KTGTANTWWWANTACAM | YP_208372.1\|NGO1308 | - | 53 | 69 | 8.06e-05 | 0.967 | GTGTTTTTTCATTTCAA |
| KTGTANTWWWANTACAM | YP_208194.1\|NGO1115 | + | 124 | 140 | 8.06e-05 | 0.967 | TTGCATTATAAGTACGT |
| KTGTANTWWWANTACAM | YP_208254.1\|NGO1182 | + | 125 | 141 | 8.06e-05 | 0.967 | TTGTATTGTTTGTTCAA |
| KTGTANTWWWANTACAM | NGO_t42\|NGO_t42 | + | 135 | 151 | 8.06e-05 | 0.967 | CGGTATTTTTTATACAC |
| KTGTANTWWWANTACAM | YP_208194.1\|NGO1115 | - | 124 | 140 | 8.06e-05 | 0.967 | ACGTACTTATAATGCAA |
| KTGTANTWWWANTACAM | YP_208254.1\|NGO1182 | - | 125 | 141 | 8.06e-05 | 0.967 | TTGAACAAACAATACAA |
| KTGTANTWWWANTACAM | NGO_t42\|NGO_t42 | - | 135 | 151 | 8.06e-05 | 0.967 | GTGTATAAAAAATACCG |
| KTGTANTWWWANTACAM | YP_208508.1\|NGO1455 | + | 164 | 180 | 8.06e-05 | 0.967 | TTGTTGTAAAATTTCAG |
| KTGTANTWWWANTACAM | YP_208038.1\|rpmE2 | + | 165 | 181 | 8.06e-05 | 0.967 | TTGTTATGTTATAACAC |
| KTGTANTWWWANTACAM | YP_208508.1\|NGO1455 | - | 164 | 180 | 8.06e-05 | 0.967 | CTGAAATTTTACAACAA |
| KTGTANTWWWANTACAM | YP_208038.1\|rpmE2 | - | 165 | 181 | 8.06e-05 | 0.967 | GTGTTATAACATAACAA |
| KTGTANTWWWANTACAM | YP_208509.1\|NGO1456 | + | 279 | 295 | 8.06e-05 | 0.967 | CTGAAATTTTACAACAA |
| KTGTANTWWWANTACAM | YP_208509.1\|NGO1456 | - | 279 | 295 | 8.06e-05 | 0.967 | TTGTTGTAAAATTTCAG |
| KTGTANTWWWANTACAM | YP_208797.1\|NGO1765 | + | 792 | 808 | 8.06e-05 | 0.967 | GTGTAAAATCATCACAA |
| KTGTANTWWWANTACAM | YP_208797.1\|NGO1765 | - | 792 | 808 | 8.06e-05 | 0.967 | TTGTGATGATTTTACAC |
| KTGTANTWWWANTACAM | YP_208914.1\|NGO1881 | + | 943 | 959 | 8.06e-05 | 0.967 | CTGTAATTTTGCAACAA |
| KTGTANTWWWANTACAM | YP_208914.1\|NGO1881 | - | 943 | 959 | 8.06e-05 | 0.967 | TTGTTGCAAAATTACAG |
| KTGTANTWWWANTACAM | YP_207523.1\|NGO0367 | + | 8 | 24 | 9.21e-05 | 0.967 | TTGTAATCAAAATTAAC |
| KTGTANTWWWANTACAM | YP_208275.1\|NGO1204 | + | 10 | 26 | 9.21e-05 | 0.967 | TTGTTATTATATAACAT |
| KTGTANTWWWANTACAM | YP_207523.1\|NGO0367 | - | 8 | 24 | 9.21e-05 | 0.967 | GTTAATTTTGATTACAA |
| KTGTANTWWWANTACAM | YP_208275.1\|NGO1204 | - | 10 | 26 | 9.21e-05 | 0.967 | ATGTTATATAATAACAA |
| KTGTANTWWWANTACAM | YP_208423.1\|NGO1363 | + | 49 | 65 | 9.21e-05 | 0.967 | TTTTATGAATACTACAT |
| KTGTANTWWWANTACAM | YP_208535.1\|NGO1485 | + | 51 | 67 | 9.21e-05 | 0.967 | GTCTAATGAAAATTCAA |
| KTGTANTWWWANTACAM | YP_208427.1\|NGO1367 | + | 62 | 78 | 9.21e-05 | 0.967 | TTGTAAGCAAACTAAAA |
| KTGTANTWWWANTACAM | YP_208423.1\|NGO1363 | - | 49 | 65 | 9.21e-05 | 0.967 | ATGTAGTATTCATAAAA |
| KTGTANTWWWANTACAM | YP_208535.1\|NGO1485 | - | 51 | 67 | 9.21e-05 | 0.967 | TTGAATTTTCATTAGAC |
| KTGTANTWWWANTACAM | YP_207980.1\|NGO0867 | + | 74 | 90 | 9.21e-05 | 0.967 | GTGTGATTAGATTAAAA |
| KTGTANTWWWANTACAM | YP_208427.1\|NGO1367 | - | 62 | 78 | 9.21e-05 | 0.967 | TTTTAGTTTGCTTACAA |
| KTGTANTWWWANTACAM | YP_208771.1\|NGO1738 | + | 87 | 103 | 9.21e-05 | 0.967 | TTTTATTTTAACCACAG |
| KTGTANTWWWANTACAM | YP_207980.1\|NGO0867 | - | 74 | 90 | 9.21e-05 | 0.967 | TTTTAATCTAATCACAC |
| KTGTANTWWWANTACAM | YP_208771.1\|NGO1738 | - | 87 | 103 | 9.21e-05 | 0.967 | CTGTGGTTAAAATAAAA |
| KTGTANTWWWANTACAM | YP_208129.1\|NGO1045 | + | 105 | 121 | 9.21e-05 | 0.967 | TTGAATTTAAAGTATAT |
| KTGTANTWWWANTACAM | YP_208080.1\|NGO0982 | + | 118 | 134 | 9.21e-05 | 0.967 | TTGTTATTTTATTGCAG |
| KTGTANTWWWANTACAM | YP_207990.1\|NGO0878 | + | 118 | 134 | 9.21e-05 | 0.967 | TTTTACAAAGAATACAA |
| KTGTANTWWWANTACAM | YP_208129.1\|NGO1045 | - | 105 | 121 | 9.21e-05 | 0.967 | ATATACTTTAAATTCAA |
| KTGTANTWWWANTACAM | YP_208080.1\|NGO0982 | - | 118 | 134 | 9.21e-05 | 0.967 | CTGCAATAAAATAACAA |
| KTGTANTWWWANTACAM | YP_207990.1\|NGO0878 | - | 118 | 134 | 9.21e-05 | 0.967 | TTGTATTCTTTGTAAAA |
| KTGTANTWWWANTACAM | YP_208130.1\|NGO1046 | + | 139 | 155 | 9.21e-05 | 0.967 | ATATACTTTAAATTCAA |
| KTGTANTWWWANTACAM | YP_208130.1\|NGO1046 | - | 139 | 155 | 9.21e-05 | 0.967 | TTGAATTTAAAGTATAT |
| KTGTANTWWWANTACAM | YP_208428.1\|NGO1368 | + | 209 | 225 | 9.21e-05 | 0.967 | TTTTAGTTTGCTTACAA |
| KTGTANTWWWANTACAM | YP_208428.1\|NGO1368 | - | 209 | 225 | 9.21e-05 | 0.967 | TTGTAAGCAAACTAAAA |
| KTGTANTWWWANTACAM | YP_208359.1\|NGO1293 | + | 252 | 268 | 9.21e-05 | 0.967 | AAGTAATTAAATTTCAA |
| KTGTANTWWWANTACAM | YP_208359.1\|NGO1293 | - | 252 | 268 | 9.21e-05 | 0.967 | TTGAAATTTAATTACTT |
| KTGTANTWWWANTACAM | YP_208276.1\|NGO1205 | + | 367 | 383 | 9.21e-05 | 0.967 | ATGTTATATAATAACAA |
| KTGTANTWWWANTACAM | YP_208276.1\|NGO1205 | - | 367 | 383 | 9.21e-05 | 0.967 | TTGTTATTATATAACAT |
| KTGTANTWWWANTACAM | YP_207584.1\|NGO0429 | + | 820 | 836 | 9.21e-05 | 0.967 | TTGGAATTTTATTACTG |
| KTGTANTWWWANTACAM | YP_208698.1\|NGO1659 | + | 834 | 850 | 9.21e-05 | 0.967 | CTGTATTTTAAAGCCAA |
| KTGTANTWWWANTACAM | YP_207584.1\|NGO0429 | - | 820 | 836 | 9.21e-05 | 0.967 | CAGTAATAAAATTCCAA |
| KTGTANTWWWANTACAM | YP_208698.1\|NGO1659 | - | 834 | 850 | 9.21e-05 | 0.967 | TTGGCTTTAAAATACAG |
| KTGTANTWWWANTACAM | YP_207981.1\|NGO0868 | + | 850 | 866 | 9.21e-05 | 0.967 | TTTTAATCTAATCACAC |
| KTGTANTWWWANTACAM | YP_207981.1\|NGO0868 | - | 850 | 866 | 9.21e-05 | 0.967 | GTGTGATTAGATTAAAA |
| KTGTANTWWWANTACAM | YP_207448.1\|NGO0288 | + | 65 | 81 | 9.47e-05 | 0.967 | ATGTAATATAATTCTAA |
| KTGTANTWWWANTACAM | YP_207292.1\|NGO0120 | + | 74 | 90 | 9.47e-05 | 0.967 | CTTTTATTTAATTACAA |
| KTGTANTWWWANTACAM | YP_207448.1\|NGO0288 | - | 65 | 81 | 9.47e-05 | 0.967 | TTAGAATTATATTACAT |
| KTGTANTWWWANTACAM | YP_207292.1\|NGO0120 | - | 74 | 90 | 9.47e-05 | 0.967 | TTGTAATTAAATAAAAG |
| KTGTANTWWWANTACAM | YP_208547.1\|NGO1497 | + | 245 | 261 | 9.47e-05 | 0.967 | ATTTACTAAAAATATAA |
| KTGTANTWWWANTACAM | YP_208547.1\|NGO1497 | - | 245 | 261 | 9.47e-05 | 0.967 | TTATATTTTTAGTAAAT |
| KTGTANTWWWANTACAM | YP_208490.1\|NGO1435 | + | 331 | 347 | 9.47e-05 | 0.967 | CTGTAATTTTAATATTA |
| KTGTANTWWWANTACAM | YP_208490.1\|NGO1435 | - | 331 | 347 | 9.47e-05 | 0.967 | TAATATTAAAATTACAG |
| KTGTANTWWWANTACAM | YP_208911.1\|NGO18781 | + | 589 | 605 | 9.49e-05 | 0.967 | TTTTATTATTAATAAAT |
| KTGTANTWWWANTACAM | YP_208911.1\|NGO18781 | - | 589 | 605 | 9.49e-05 | 0.967 | ATTTATTAATAATAAAA |

**References**

1. Grant CE, Bailey TL, Noble WS (2011) FIMO: scanning for occurrences of a given motif. Bioinformatics 27: 1017-1018.

2. Antunes A, Golfieri G, Ferlicca F, Giuliani MM, Scarlato V, et al. (2015) HexR Controls Glucose-Responsive Genes and Central Carbon Metabolism in Neisseria meningitidis. J Bacteriol 198: 644-654.
