# Supplementary material for: Transcriptional regulation of a gonococcal gene encoding a virulence factor (L-lactate permease)
Source: PLoS Pathog. 2019 Dec 20;15(12):e1008233. doi: 10.1371/journal.ppat.1008233 (PMC6957213; doi:10.1371/journal.ppat.1008233)
Supplement: S4 Appendix — (DOCX) [file ppat.1008233.s016.docx]

**S4. Appendix. Effect of the *ptsK* allele deletion on the glucose-mediated repression of *lctP.***

**
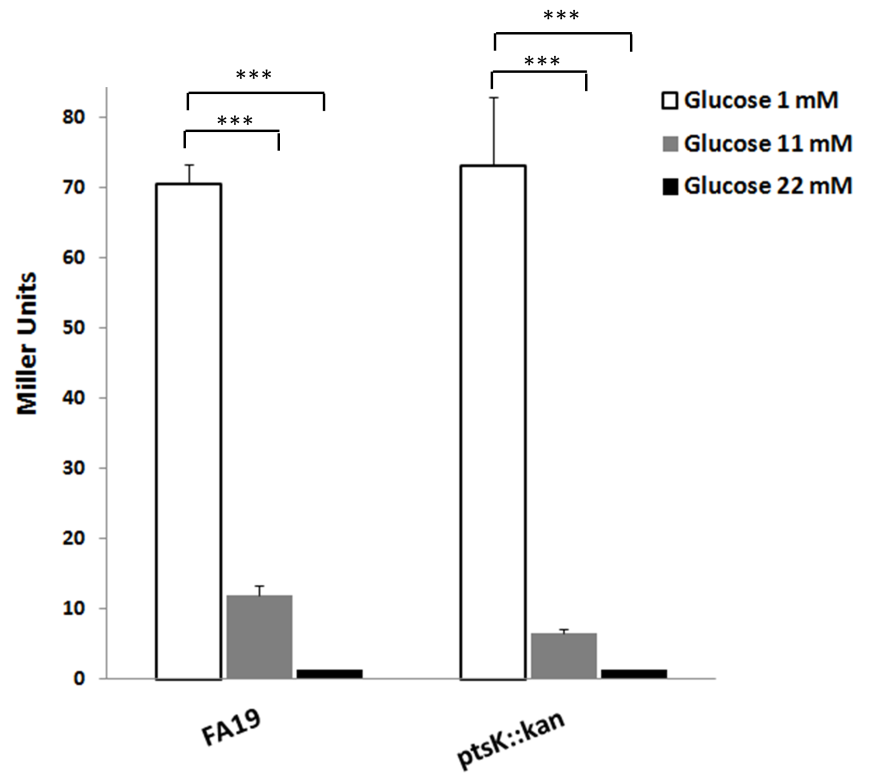
**

FA19 reporter strain JC28 and isogenic mutant JC41 (*ptsK::kan*) containing an *lctP*-*lacZ* fusion in vector pLES94-*lctP* were grown to stationary phase on GC broth supplemented with 1, 11 or 22 mM D-glucose and a fix concentration of 3 mM L-lactate each. β-galactosidase was expressed from the *lctP* transcriptional and translational signals and its activity was determined in Miller units. Data are presented as the mean (bar) plus the standard error of the mean (error bar) of 3 biological samples and two technical replicates each. *** represents significant statistical differences at p<0.001 as determined by an ANOVA test and Tukey’s posttest.

**Construction of the *ptsK* insertional mutation**

To construct a *ptsK* insertional mutation a PCR fragment encoding the *ptsK* allele was amplified with primers EcoR-ptsk-F (GAGAATTCGCCGTCTGAAGCCCAGTATCTCCGTCCG) and Sph-ptsK-R (ATGCATGCGCGCTTGGGCAATGTCTATG) and ligated into EcoRI-SphI-digested pUC19 to create pUC19-*ptsK*. Then the kanamycin resistance cassette from HincII-digested pUC4K [[1](#_ENREF_1)] was blunt-end ligated at the HincII site of pUC19-*ptsK* to generate pUC19-*ptsK*::*kan*. Next, vector pUC19-*ptsK*::*kan* was linearized with EcoRI and used for transformation of JC28 reporter strain to generate JC41. Transformant JC41 was selected on GC-agar plates containing kanamycin and disruption of the *ptsK* allele was confirmed by PCR with primers pacptsK-F (TATCTTAATTAAATGCCCAGTATCTCCGTCCG) and pmeptsK-R (ATGTTTAAACGCGCTTGGGCAATGTCTATG).

**References**

1. Vieira J, Messing J (1982) The pUC plasmids, an M13mp7-derived system for insertion mutagenesis and sequencing with synthetic universal primers. Gene 19: 259-268.
